# Melatonin seed priming: A climate‐smart, green strategy to enhance abiotic stress tolerance in plants

**DOI:** 10.1111/jipb.70291

**Published:** 2026-05-24

**Authors:** Ali Raza, Yiran Li, Chunli Guo, Erna Karalija, Evgenios Agathokleous, Meng Jiang, Jie Zhou, Vasileios Fotopoulos, Zhangli Hu

**Affiliations:** ^1^ Guangdong Key Laboratory of Plant Epigenetics, College of Life Sciences and Oceanography Shenzhen University Shenzhen 518060 China; ^2^ Shenzhen Engineering Laboratory for Marine Algal Biotechnology, Guangdong Technology Research Center for Marine Algal Biotechnology, Longhua Innovation Institute for Biotechnology, College of Life Sciences and Oceanography Shenzhen University Shenzhen 518060 China; ^3^ Faculty of Science, Laboratory for Plant Physiology and Molecular Biology University of Sarajevo Sarajevo 71000 Bosnia and Herzegovina; ^4^ Key Laboratory of Agrometeorology of Jiangsu Province, School of Ecology and Applied Meteorology Nanjing University of Information Science & Technology (NUIST) Nanjing 210044 China; ^5^ Research Center for Global Changes and Ecosystem Carbon Sequestration & Mitigation, School of Ecology and Applied Meteorology Nanjing University of Information Science & Technology (NUIST) Nanjing 210044 China; ^6^ Hainan Institute, Yazhou Bay Science and Technology City Zhejiang University Sanya 572025 China; ^7^ National Key Laboratory of Rice Biology and Breeding, The Advanced Seed Institute Zhejiang University Hangzhou 310058 China; ^8^ Department of Horticulture, Zhejiang Provincial Key Laboratory of Horticultural Crop Quality Regulation Zhejiang University Hangzhou 310058 China; ^9^ Department of Agricultural Sciences, Biotechnology & Food Science Cyprus University of Technology Lemesos 3036 Cyprus; ^10^ Guangdong Provincial Key Laboratory of Functional Substances in Medicinal Resources and Healthcare Products, School of Life Sciences and Food Engineering Hanshan Normal University Chaozhou 521041 China; ^11^ Hunan Innovation Center for Synthetic Biomanufacturing Industry Hunan University of Arts and Science Changde 415000 China

**Keywords:** biostimulant, hormonal crosstalk, hydropriming, molecular breeding, nanoformulations, stress memory, stress‐smart seeds

## Abstract

Enhancing crop tolerance to multiple abiotic stresses is critical for achieving sustainable agriculture. Targeted seed‐stage interventions using natural signaling compounds (e.g., melatonin) provide a unique opportunity to establish early stress tolerance that can persist through the critical seed‐to‐seedling transition. Melatonin seed priming (MSP) is rapidly emerging as a green and climate‐smart strategy for enhancing plant stress tolerance. MSP triggers defensive molecular, biochemical, and physiological reprogramming during germination, thereby improving plant performance under subsequent stress conditions. This review synthesizes recent mechanistic insights into how MSP confers stress tolerance across diverse species by modulating redox signaling, hormonal homeostasis, and stress‐related gene networks. We elucidate the synergistic potential of MSP when combined with nanoformulations, other priming agents, or beneficial microbes. We also discuss its crosstalk with key signaling pathways to better understand the tolerance mechanisms. Furthermore, we propose a forward‐looking strategy that integrates omics, genome editing, speed breeding, and molecular phenotyping methods to improve MSP applications for the development of stress‐smart crops. Despite its potential, MSP still faces multiple challenges, including species‐specific responses, dosage variability, limited post‐priming seed storage stability, and a lack of field‐scale validation. Addressing these bottlenecks through high‐throughput screening, epigenetic memory assessment, and optimized delivery systems will be essential to fully harness the practical potential of MSP as a sustainable and green approach for future agriculture.

## Graphical abstract

This review synthesizes how melatonin seed priming preconditions seeds to enhance tolerance against diverse abiotic stresses. It highlights the underlying mechanisms and proposes an integrative roadmap of advanced molecular and breeding tools to design next‐generation, stress‐smart plants.

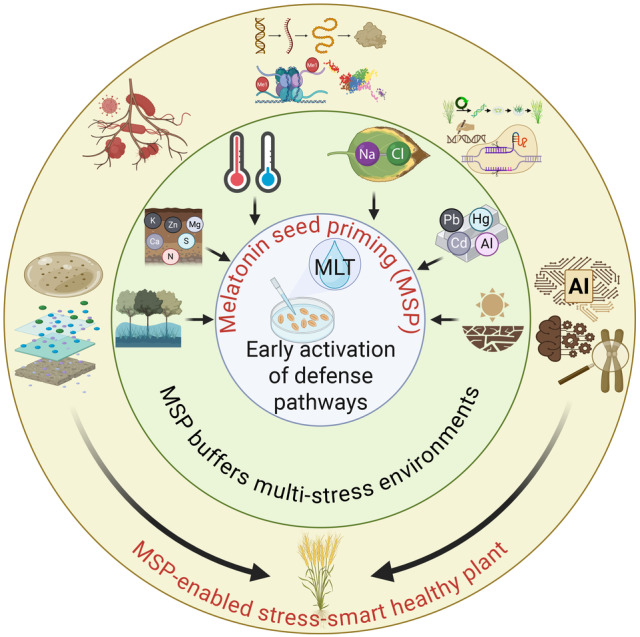

## STRESS‐SMART SEEDS: A WAY FORWARD FOR CLIMATE‐SMART AGRICULTURE

Seeds form the foundation of agriculture, carrying the genetic, physiological, and biochemical heritage that determines plant vigor and stress tolerance. Germination and early seedling development are considered the most vulnerable phases in the crop life cycle, during which abiotic stress can cause irreparable losses in plant stand, growth potential, and yield. Thus, seeds are not merely passive carriers of genetic materials, but active platforms for pre‐emptive stress management (Forni and Borromeo, 2023; Pagano et al., 2023; Cañizares et al., 2025). Hence, seeds are not only key targets for interventions to enhance crop performance but also strategic entry points for developing climate‐smart plants, which set the stage for green innovations such as seed priming, which pre‐condition seeds to withstand multiple climatic stresses.

Abiotic stresses, including air pollution, drought, salinity, high/low temperature, flooding, nutrient imbalances, or heavy metals, severely constrain global agricultural productivity. While these stresses are frequently studied at the vegetative or reproductive stages, seed‐stage stress tolerance remains an underestimated, yet critical factor in plant stress tolerance. As climate unpredictability intensifies, developing unique strategies to improve early‐stage plant performance is ever more critical (Savvides et al., 2016; Tripathi et al., 2024; Raza et al., 2025a; Ateeq et al., 2026; Wang et al., 2026b). Among such strategies, seed priming has emerged as an efficient, non‐transgenic, green method to shield crops against multiple stresses (Cañizares et al., 2025; Gohari et al., 2026; Pasquali Medici de Biron et al., 2026).

Seed priming is a powerful technique that improves germination, seedling establishment, and overall crop performance under stress conditions (Forni and Borromeo, 2023; Pagano et al., 2023; Cañizares et al., 2025; Gohari et al., 2026). This pre‐sowing intervention prepares seeds for rapid and synchronized germination by activating metabolism without allowing radicle emergence. Adjusting this early metabolic “wake‐up call” effectively trains plants to mount a pre‐emptive defense against multiple stresses simultaneously. By effectively “training” seeds before they confront the field environment, priming can shift stress responses from reactive to proactive, giving plants a critical head start. Such preparatory steps may help consistently bridge the yield gap under climate uncertainty. Priming is widely used in sustainable agriculture to boost stress tolerance without genetic modification; it can mitigate the effects of abiotic stresses by jump‐starting physio‐biochemical defenses before stress occurs (Savvides et al., 2016; Rajora et al., 2022; Forni and Borromeo, 2023; Pagano et al., 2023; Tripathi et al., 2024; Cañizares et al., 2025; Pasquali Medici de Biron et al., 2026). Notably, priming can reduce the need for intensive post‐emergence management. Yet, challenges remain in optimizing protocols, ensuring post‐priming stability, and validating performance under natural field conditions (addressed in detail in section “A CRITICAL ASSESSMENT OF MSP: LIMITATIONS, INCONSISTENCIES, AND KNOWLEDGE GAPS”).

Melatonin (MLT, N‐acetyl‐5‐methoxytryptamine) has gained great interest as a priming agent (Rajora et al., 2022; Zhu et al., 2025b). Among various priming agents, MLT is uniquely suitable for seed‐stage interventions due to its dual role as a potent antioxidant that directly scavenges reactive oxygen species (ROS) and as a signaling molecule that can prime stress‐responsive pathways without toxicity, even at elevated levels (Wang et al., 2018, 2024b; Agathokleous et al., 2021; Sun et al., 2021, 2025; Rajora et al., 2022; Zeng et al., 2022b; Ahmad et al., 2023; Colombage et al., 2023; Zhu et al., 2025b). Unlike synthetic priming agents, MLT is a naturally occurring, non‐toxic, multifunctional, low‐cost, and ecologically benign compound, making it exceptionally attractive for sustainable agriculture. Exogenous MLT enhances seed germination and root growth, boosts antioxidant activities, and preserves photosynthetic apparatus under stress (Agathokleous et al., 2021; Zhu et al., 2025b). Although there are many reviews on MLT's roles, they mostly focused on foliar or exogenous applications in established plants (Wang et al., 2018, 2024b; Sun et al., 2021; Raza et al., 2022, 2026a; Zeng et al., 2022b; Ahmad et al., 2023; Colombage et al., 2023; Pan et al., 2023; Huang et al., 2024; Jindal et al., 2024; Rachappanavar, 2025). In contrast, MLT application through seed priming still remains underexplored relative to its practical potential. A prior review mostly classified MLT priming along with its exogenous effects across four stresses and general mechanisms in established plants (Rajora et al., 2022). However, this review does not deliver detailed stress‐specific MLT‐enabled priming outcomes, integration with biotechnological/breeding tools, or forward‐looking perspectives on synergies and bottlenecks. Therefore, the present review focuses on MLT seed priming (hereafter MSP) and delivers a seed‐centric synthesis of mechanisms, advances, and applications across single and combined abiotic stresses.

To ensure a comprehensive and transparent synthesis, we conducted a systematic literature search using the Web of Science, Google Scholar, and Scopus databases. We used keyword combinations including “melatonin seed priming”, “melatonin priming”, and “seed priming with melatonin”, in combination with specific abiotic stress terms such as “drought, salt/salinity, temperature/heat/cold, heavy metals, waterlogging/flooding, nutrient deficiency, and combined stresses” (e.g., melatonin seed priming + stress name). No limits were applied on publication year, journal, or crop species to maximize coverage. Titles and abstracts of retrieved articles were screened for relevance, followed by full‐text assessment. Studies were included if they involved MLT applied as a seed priming treatment (soaking or coating) with abiotic stress tolerance outcomes.

MLT delivered at the seed stage can pre‐activate stress defense pathways before germination and contribute toward delivering next‐generation, stress‐smart seeds (in other words, seeds that can tolerate/adapt to stress conditions). This review critically evaluates MSP as a rapidly emerging, sustainable seed‐based strategy to enhance plant tolerance to abiotic stress. We (i) highlight mechanisms and outcomes in a stress‐specific manner, (ii) identify synergies, bottlenecks, and gaps, (iii) navigate integration with omics and molecular breeding tools, and (iv) suggest underexplored directions to fully harness the power of MSP for stress‐smart seeds.

## SEED PRIMING WITH MELATONIN: A BRIEF APPRAISAL ON CONCEPTS AND TECHNIQUES

Seed priming involves the controlled hydration of seeds and encompasses various methods. Depending on the treatment medium, priming can be categorized into several types: (i) hydropriming, where seeds are soaked in water or MLT; (ii) osmopriming using osmotic solutions (e.g., PEG or salts); (iii) halopriming applying inorganic salts; (iv) nutripriming with nutrient solutions; (v) hormopriming or hormonal priming involving plant growth regulators such as phytohormones or MLT; (vi) biopriming including plant growth‐promoting bacteria; and (vii) nanopriming with nanoparticles; and (viii) solid‐matrix priming or coating‐assisted priming, where seeds are encapsulated or coated with functionalized hydrocolloid matrices (Forni and Borromeo, 2023; Pagano et al., 2023; Tripathi et al., 2024; Athanasiou et al., 2025; Cañizares et al., 2025). MSP falls within hormopriming or hydropriming, where seeds are soaked in an aqueous MLT solution for several hours, and then air‐dried to their original moisture content before sowing. For instance, in tartary buckwheat (*Fagopyrum tataricum* Gaertn.), a 3 h soak in MLT (50 μM L^−1^) achieved nearly 100% germination under salinity stress (Zhu et al., 2025b).

The efficacy of MSP is highly context‐dependent, which requires optimization of the MLT concentration and the soaking duration based on the species, seed type, and the target abiotic stresses (Savvides et al., 2016; Agathokleous et al., 2021; Rajora et al., 2022; Forni and Borromeo, 2023; Tripathi et al., 2024; Gohari et al., 2024a; Cañizares et al., 2025; Zhu et al., 2025b). This optimization is essential, as sub‐optimal or excessive doses can limit efficacy or even inhibit germination.

**Figure 1:**
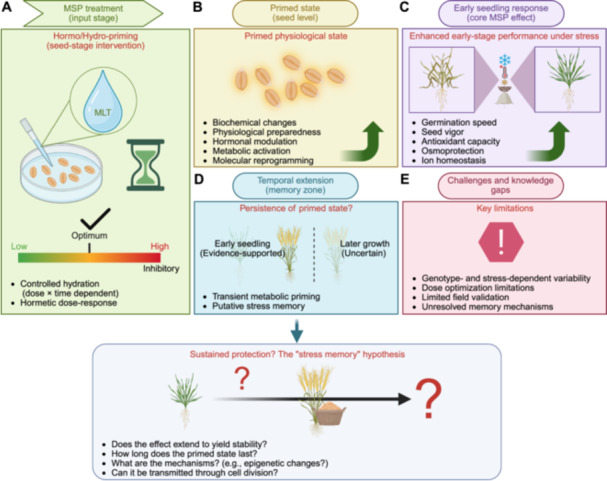
Conceptual model of melatonin (MLT) seed priming for developing stress‐smart seedlings **(A)** MLT seed priming (MSP) is performed as hormo‐ or hydro‐priming by soaking seeds in an optimized melatonin solution for a controlled duration, followed by drying back to the original moisture content. Efficacy is highly dose‐ and time‐dependent, showing a hormetic response where low to moderate doses are beneficial, while high doses can be inhibitory. **(B)** This treatment induces a primed state characterized by improved reprogramming of diverse mechanisms. **(C)** Under subsequent abiotic stress, MPS‐primed seeds show faster and more uniform germination, improved seedling vigor, and enhanced stress tolerance mechanisms. **(D)** The persistence of this primed state into later growth and yield remains uncertain, an open question, and is a working hypothesis that requires in‐depth validation. **(E)** This panel highlights the major challenges hindering the translation of MSP from laboratory to field. Created in https://BioRender.com.

Compared with other priming strategies, seed priming is considered a cost‐effective and eco‐friendly approach to enhance abiotic stress tolerance because it requires only small volumes of bioactive compounds and minimal infrastructure (Savvides et al., 2016; Forni and Borromeo, 2023; Cañizares et al., 2025). MLT priming, in particular, is quite helpful due to its pleiotropic functions, that is, it enhances germination, strengthens antioxidant defense systems, and modulates stress‐responsive hormone signaling (Rajora et al., 2022; Zhu et al., 2025b). Furthermore, the benefits of this initial seed treatment are frequently not limited to the moment of germination. The priming process can induce a form of physiological stress memory, a working hypothesis where molecular and physio‐biochemical changes initiated during priming persist into early seedling growth (Figure 1). This primed state can lead to enhanced performance under stress conditions during subsequent developmental stages (Rajora et al., 2022; Zhu et al., 2025b). The persistence of these effects into later growth stages and the potential epigenetic stress memory remain open questions that demand field validation (Hilker and Schmülling, 2019; Dobránszki et al., 2025).

**Figure 2:**
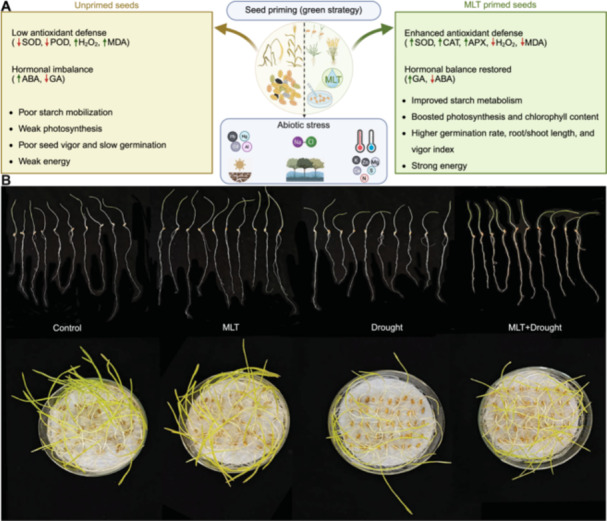
Melatonin seed priming activates early stress defense mechanisms **(A)** Conceptual model illustrating how melatonin (MLT)‐primed seeds reprogram antioxidant systems and hormonal pathways during germination under abiotic stress. Compared to unprimed seeds (left), MLT‐primed seeds (right) show improved physio‐biochemical traits, which enable seeds to overcome oxidative and hormonal stress barriers during germination and ultimately result in vigorous seedling establishment and stress tolerance. These findings are based on wheat (Guo et al., 2022) and maize (Cao et al., 2019) seeds' priming under drought and cold stress conditions. Upward green arrows (↑) indicate increased, accumulation, or upregulated mechanisms and the downward red arrows (↓) indicate decreased, suppressed, or downregulated mechanisms. **(B)** Wheat (triticale) seedling responses to drought stress with or without MLT seed priming showed improved germination and physiological traits. This image was adapted from Guo et al. (2022) under the terms of the Creative Commons Attribution License (CC BY International License; https://creativecommons.org/licenses/by/4.0/). Created with the aid of https://BioRender.com. ABA, abscisic acid; CAT, catalase; GA, gibberellic acid; H_2_O_2_, hydrogen peroxide; MDA, malondialdehyde; POD, peroxidase; and SOD, superoxide dismutase.

Thus, MSP not only improves early development but can also impart improved tolerance during subsequent growth phases, though the continuity of these effects depends on the species, dose, and stress type. From a practical perspective, MSP requires far less MLT than repeated foliar applications, thereby reducing both costs and the environmental footprint. Seed treatments are straightforward to integrate into present farming practices, which require only simple infrastructure such as soaking tanks or seed‐coating formulations. In Figure 2, we conceptually illustrated how MSPs contribute to early metabolic reprogramming, hormonal homeostasis, and the antioxidative machinery, which enable superior seedling establishment under stress conditions.

## HOW DOES MSP BOOST TOLERANCE AGAINST ABIOTIC STRESSES? SEED‐LEVEL MECHANISTIC INSIGHTS

**Figure 3:**
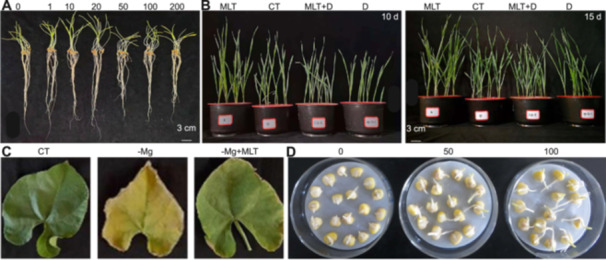
Phenotypic traits of different plants harvested with melatonin (MLT) seed priming **(A)** Germination of MLT (μM)‐primed triticale wheat seeds after 7 d. **(B)** Phenotype of triticale seedling in pot cultivation under drought stress and with MLT treatment. **(C)** Common bean leaves cultivated in hydroponics with a magnesium (Mg)‐sufficient solution (CT), a Mg‐deficient solution (−Mg), and a Mg‐deficient solution with MLT‐primed seeds (−Mg + MLT). **(D)** Morphology characteristics of maize seeds after 9 d of germination with various MLT concentrations (μM) for 24 h. These images were adapted and text‐edited from Open Access publications under Creative Commons Attribution License (CC BY International License; https://creativecommons.org/licenses/by/4.0/). Panels A and B were taken from (Guo et al., 2022); panel C was taken from (Bouzidi and Krouma, 2025); and panel D was taken from (Cao et al., 2019).

**Figure 4:**
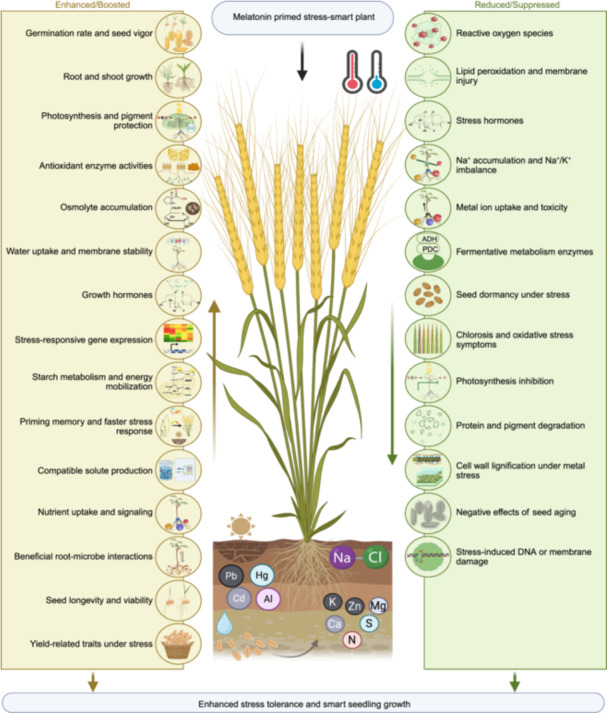
Enhanced or reduced mechanisms by which melatonin seed priming (MSP) enhances abiotic stress tolerance, seed germination, and seedling growth These mechanisms are discussed in detail in stress‐specific sections herein. Created in https://BioRender.com.

As a seed‐stage stress conditioning approach, MSP has been shown to mitigate abiotic stresses. Representative images demonstrate that MSP‐mediated protection results in visible improvements in germination, root/shoot biomass, and overall plant health across diverse species and stress conditions (Figure 3). In the subsections below, we summarize evidence across major stress types and the representative studies summarized in Table 1. Building upon these findings, Figure 4 proposes the major mechanisms of MSP‐driven stress tolerance in plants.

**Table 1:** Representative studies highlighting the protective role of melatonin seed priming under different abiotic stresses

Plant species	Stress condition	MSP (dose, duration, and category)	Final growth condition	Key findings	Stress tolerance mechanisms	Reference
Drought						
Wheat	2% PEG 6000; 7 d	100 μM; 12 h (Hydro)	Petri dish	↑ Root hydraulic conductivity ↑ Root length ↑ Seedling growth	↑ Expression of aquaporin genes **↑** ROS detoxification ↑ Antioxidant enzyme activities Maintenance of K^+^ ion balance in roots and shoots	Fu et al. (2024)
Maize	PEG‐6000; 15 d	250, 500, and 1,000 μM; 6 h (Hydro)	Polyethylene boxes	↑ Seed germination and shoot/root growth at 500 μM ↑ Chl content and stomatal traits ↓ H_2_O_2_, MDA, and EL	↑ SOD, CAT, POD, AsA, and GSH activities ↑ ROS mitigation and osmotic balance Stabilized chloroplasts and cell walls	Muhammad et al. (2023)
Rapeseed	35%–40% FC; till maturity	500 μM; 6 h (Hydro)	Pot and field	↑ Proline accumulation ↓ Yield loss and quality deterioration Field validation of priming effects	↑ ROS scavenging through enzymatic antioxidant activation ↑ Osmoprotection via proline accumulation **↑** Yield stabilization	Khan et al. (2020)
Safflower	85% of the soil moisture; flowering to harvest	0.1 and 0.5 mM; 6 h (Hydro)	Field	↓ Lipid peroxidation Fresh seeds responded better than aged seeds at 0.1–0.5 mM Dose‐dependent responses observed	**↑** Antioxidant enzyme activities ↑ Membrane protection and integrity	Heshmati et al. (2021)
Triticale‐wheat	10% PEG‐6000; 7 d	20 μM; 24 h (Hydro)	Petri dish and pot	↑ Germination rate ↑ Leaf area and net photosynthesis ↓ ROS and MDA	↑ Antioxidant enzyme activation (SOD and POD) ↑ Energy metabolism and water relations	Guo et al. (2022)
Wheat	35% FC; sowing to harvest	2 mg L^−1^; 16 h (Hydro)	Pot	↑ Germination rate ↑ TSP, proline, soluble sugars, and GB ↑ Biomass and pigment levels	↑ Accumulation of osmolytes and compatible solutes ↑ ROS detoxification and membrane stabilization ↑ Genotype‐specific stress response	Shaheen et al. (2024)
Sorghum	18% PEG‐6000; 7 d	50, 100, 200, and 300 μM; 7 d (Hydro)	Petri dish	↑ Germination in aged rice, barley, and sorghum ↑ PEG‐induced drought in sorghum at 200 μM	↑ Priming rescued seed vigor under stress ↑ Cell division and early seedling growth	García‐Cánovas et al. (2024)
Peanut	20% of previous day's transpiration until transpiration < 30% of control; 15 d	50 μM; 12 h (Hydro)	Pot	↑ CO_2_ assimilation and transpiration ↑ Root water uptake ↑ Membrane stability ↓ ROS	↑ Vegetative growth and physiological performance Sensitized drought responses in tolerant vs. sensitive genotypes	de Camargo Santos et al. (2024)
Pearl millet	40% water‐holding capacity; 35 d	120 μM; 24 h (Hydro)	Pot	↑ Proline, CAT, SOD, POD, GR, and GPX activities ↓ MDA and H_2_O_2_ levels Gene expression linked to antioxidant responses	↑ Expression of drought‐responsive antioxidant genes ↑ Oxidative stress mitigation and osmotic regulation	Awan et al. (2024)
Rice	Withholding Hoagland's solution; 6 d	60, 90, 120, 150, and 180 ppm; 3 d (Hydro)	Pot	↑ Root system architecture and Chl at 120 ppm ↑ Antioxidant enzyme activity ↑ Seedling biomass	↑ Photosynthetic machinery ↑ ROS scavenging and protein metabolism	Tyagi et al. (2025)
Cotton	Withholding the irrigation; 4 d	10 μM; 24 h (Hydro)	Pot	↑ Drought symptoms (wilting or stunted growth) ↑ Endogenous MLT ↑ Chl, photosynthesis, and sugar metabolism ↓ ABA level	↑ ABA‐independent autophagy via *MAPK6*, *SnRK2.6*, and KIN10 signaling ↑ *ATG8* and *RAPTOR1* expression to stimulate autophagy ↑ T6P levels are regulated by trehalose gene expression MLT acted upstream of autophagy‐regulating kinase cascades	Supriya et al. (2024)
Salinity						
Buckwheat	100 mmol L^−1^ NaCl; 7 d	50 μM; 3 h (Hydro)	Petri dish	↑ Germination ↑ Seedling/root length and fresh weight ↑ Seed vigor across storage conditions	↑ Antioxidant enzyme activity ↑ Seedling growth and vigor ↓ Oxidative damage during storage	Zhu et al. (2025b)
Maize	150 and 300 mM NaCl; 8 d	1,000 μM; 20 h (Hydro)	Petri dish	↑ Salt‐induced reductions in growth ↑ Chl, K^+^, and carotenoids ↓ ROS, Na^+^, ABA, MDA, and H_2_O_2_	↓ Oxidative and ionic stress ↑ K^+^/Na^+^ balance and osmotic adjustment ↑ Antioxidant defenses	Ismaeil et al. (2025)
Halophytes (*Portulaca oleracea* and *Zygophyllum simplex*)	100, 200, 300, 400, and 500 mM NaCl; ~20 d	5, 100, and 500 μM; 24 h (Hydro)	Petri dish	↑ Halophyte germination ↑ Relieved dormancy at 5 and 100 μM ↑ Seedling growth and pigment levels	↑ Seed recovery and seedling vigor ↑ Chl biosynthesis Low doses are most effective	Hussain et al. (2024)
Sorghum	Mixed salts (NaCl, MgSO_4_, and CaCl_2_) 0.27, 2.5, 3:5.0, and 8.0 dS m^−1^; from sowing to harvest	100 μM; 24 h (Hydro)	Pot	↑ Growth ↑ K^+^/Na^+^ and Ca^2+^/Na^+^ ratios ↑ Macro/micronutrient uptake	↑ Ionic balance ↑ Nutrient uptake and biomass ↑ Photosynthesis	Kiremit et al. (2024)
*Suaeda corniculata*	50, 100, 200, and 300 mmol L^−1^ NaHCO_3_; 7 d	50, 100, 150, and 200 μM L^−1^; 12 h (Hydro)	Petri dish	↑ Germination at 50 μM ↑ Antioxidant enzyme activities ↑ Improved shoot/root growth ↓ MDA	↑ SOD, POD, and CAT activities ↓ Lipid peroxidation ↑ Osmotic tolerance	Zhang et al. (2024a)
*Sophora alopecuroides*	Mixed NaCl: Na_2_SO_4_ (1:1) 50, 100, 150, 200, 250, and 300 mM; throughout germination	50, and 100 μM L^−1^ (Hydro)	Cavity tray	50 μM optimal for salinity and 100 μM for alkali stress ↑ Germination, photosynthesis, and antioxidant defense	↑ Osmotic regulation and photosynthetic pigments ↑ ROS detoxification Concentration‐dependent effects	Zhang (2025)
Common bean	4, 8, 10, and 16 dS m^−1^ NaCl; until harvest	100 μM; 10 h (Hydro)	Pot	↑ Rhizobium interaction and yield ↑ K^+^/Na^+^ and proteins ↓ ROS	↑ Antioxidant enzyme systems ↑ Symbiotic N‐fixation and ion balance ↑ Strengthened stress response	Alinia et al. (2022b)
Common bean	4, 8, 10, 16, 20, and 25 dS m^−1^ NaCl; 8 and ~80 d	20, 100, and 500 μM; 10 h (Hydro)	Petri dish and pot	↑ Salt thresholds and biomass at 20 and 100 μM ↑ K^+^/Na^+^ ratio ↓ Na^+^ concentration, and MDA level	↑ Antioxidant defense systems ↑ Ionic homeostasis ↑ Dry weight and tolerance	Alinia et al. (2021)
Wheat	100 and 300 mM NaCl; 7 d	500 μM; 20 h (Hydro)	Petri dish	↑ Germination rate and seedling growth ↑ Radicle/coleoptile length and dry weight ↑ Chl, RWC, and sugar content ↓ ROS, MDA, H_2_O_2_, and Na^+^ levels	↑ Antioxidant enzymes ↑ Sucrose metabolism and amylase activity ↑ K^+^/Na^+^ balance ↑ Photosynthetic efficiency and water status	Ismaeil et al. (2024)
Sweet corn	Mixed NaCl, MgSO_4_, and CaCl_2_: 0.27, 2.50, 5.0, and 8.0 dS m^−1^; entire growing period	50, 100, and 200 μM; 24 h (Hydro)	Pot	↑ Growth and nutrient uptake at 100 μM ↑ SPAD, K^+^/Na^+^, and Ca^2+^/Na^+^ ratios	↑ Chl content ↑ Mineral uptake	Sezer et al. (2021)
Cotton	100 mM NaCl; 14 d	25, 50, and 75 μM; 24 h (Hydro)	Plastic germination box (water culture)	↑ Seedling phenotype at 25 μM ↑ Antioxidant and hormone gene's expression ↓ Photosynthesis gene's expression	Controlled photosynthetic adjustments ↑ Antioxidant and hormone signaling ↑ ROS scavenging	Zhang et al. (2021b)
*Sarcozygium xanthoxylon*, *Nitraria tangutorum*, and *Ammopiptanthus mongolicus*	Saline soil; 21 d	100, 200, and 300 μM L^−1^; 12 and 24 h (Hydro)	Cavity trays	↑ Plant vigor at 100/300 μM L^−1^ ↑ Antioxidants and proline at low MLT (100) ↓ MDA Genotype‐specific responses	↑ Root growth and osmoregulation ↑ Antioxidant enzyme activation ↑ Synergism with IAA	Zhang et al. (2024b)
Soybean	150 mM NaCl; 7 d	20, 50, 100, 200, and 300 μM L^−1^; 12 h (Hydro)	Petri dish	↑ Germination, radical length, and biomass at 50–100 μM ↑ Boosted antioxidants ↓ MDA and H_2_O_2_ levels	↑ Antioxidant defenses (SOD, CAT, POD, and APX) ↓ Oxidative stress ↑ Germination	Awan et al. (2023)
*Salvia miltiorrhiza*	70 and 100 mM NaCl; 30 d	20 μM; 2 h (Hydro)	Pot	↑ Germination energy and percentage, and plant biomass ↑ K^+^ and GA_3_ levels ↑ Antioxidants activities ↓ MDA, H_2_O_2_, ABA, and Na^+^ ↓ ABA/GA_3_ ratio ↓ Expression of *SmNCED3*/*5*, *SmRbohD*/*F*, *SmCYP76AH1*, and *SmKSL1*	↑ GA biosynthesis and ABA catabolism ↑ ROS detoxification ↑ Ionic homeostasis (K^+^/Na^+^ balance) ↓ Oxidative stress ↓ Tanshinone biosynthesis via H_2_O_2_‐mediated regulation	Li et al. (2026)
Sunflower	75 and 150 mM NaCl; 21 d	100 μM; 16 h (Hydro)	Pot	↑ Root and shoot fresh weight ↑ Antioxidant enzyme activities ↓ Electrolyte leakage ↓ MDA and H_2_O_2_ levels Genotype‐dependent responses	↑ Synergistic activation of antioxidant defenses ↑ ROS scavenging via SOD, CAT, and POD ↑ Membrane stability Genotype‐specific priming responsiveness	Zia et al. (2026)
Extreme temperature						
Waxy maize (cold)	13°C; 9 d	50, 100 μM; 12, and 24 h (Hydro)	Culture dish	↑ Seedling vigor under 13°C at 50 and 100 μM ↑ Enhanced radicle and hypocotyl length ↓ MDA and H_2_O_2_	↑ SOD, CAT, POD, and APX activity ↑ Starch metabolism ↑ Seed vigor index	Cao et al. (2019)
Mung bean (cold)	5°C; 2 d	50 μM L^−1^; 3 d (Hydro)	Petri dish	↑ Endogenous MLT levels ↑ PAL activity and phenolic content ↓ EL after rewarming	Antioxidant activity linked to phenolics ↓ Lipid peroxidation ↑ Membrane stability	Szafrańska et al. (2014)
Mung bean (cold)	5°C; 2 d	20 μM (Hydro)	Plastic box	↑ Ultrastructural protection in plastids ↑ Root elongation post‐stress ↓ Root lipid peroxidation after chilling	↓ Oxidative stress Stabilized membrane structures Maintained cell ultrastructure after rewarming	Szafrańska et al. (2013)
Maize (cold)	5°C; 14 d	50 and 500 μM; 3 h (Hydro)	n/a	↑ Seedling establishment at 5°C ↑ Metabolic preparedness at 500 μM MLT‐primed maize showed an altered seed proteome	↑ Induction of MLT‐associated proteins ↑ Expression of stress‐related proteins Proteome shift aiding chilling tolerance	Kołodziejczyk et al. (2016)
Pepper (cold)	15°C; entire germination	1, 5, 10, or 25 μM; 24 h (Hydro)	Plastic cup	↑ Germination at 1–5 μM ↑ Emergence rates ↓ MDA and H_2_O_2_ levels	↑ SOD and CAT activities ↑ Antioxidant defense ↓ Lipid peroxidation	Korkmaz et al. (2017)
Wheat (cold)	12.0°C ± 0.5°C; 7 d	500 μM; 24 h (Hydro)	Field and petri dish	↑ Germination ↓ Chloroplast damage in coleoptiles ↓ Parental MLT reduced grain weight	↑ Antioxidant enzyme activities ↑ Starch breakdown ↑ Transgenerational stress memory	Zhang et al. (2021a)
Wheat and rye (heat)	44°C; 6 h	5, 20, 50, and 100 μM; 2 h (Hydro)	Petri dish	↑ Seedling heat tolerance ↑ Carbohydrate accumulation ↓ Shoot/root inhibition at 20–100 μM	↑ CAT and POD activity ↑ Growth and enzyme function ↓ ROS, MDA, and H_2_O_2_	Kolupaev et al. (2023)
Soybean (cold + heat)	10°C and 30°C; 7 d	20, 50, 100, 200, and 300 μM L^−1^; 12 h (Hydro)	Petri dish	↑ Germination under heat and cold at 50–100 μM ↑ Radical length and biomass ↓ MDA and H_2_O_2_ levels	↑ SOD, CAT, POD, and APX activities ↑ Proline accumulation for osmoprotection ↓ Oxidative damage	Awan et al. (2023)
Tomato (cold)	15°C and 12°C; throughout the seedling emergence period	5 and 50 μM L^−1^; 48 h (Hydro)	Plug trays	↑ Seeds germinated faster under cold at 50 μM ↑ Photosynthetic efficiency ↑ Seedling growth	↑ CBF pathway for cold acclimation ↑ Polyphenols and flavonoids ↓ Lipid peroxidation and H_2_O_2_	Park et al. (2025)
Onion and leek (cold + heat)	7°C and 35°C; 21 d	5, 10, and 25 μM; 24 h (Hydro)	Petri dish	↑ Germination index at 5 μM Tryptophan had stronger effects in onion MLT did not affect the mean germination time	↑ Early stress tolerance ↓ Chilling damage to seedlings Protective against cold but not under heat	Hancı et al. (2019)
Heavy metals						
Chickpea (cadmium)	200 μM CdCl_2_; 6 d	10 μM; 12 h (Hydro)	Aqueous solution	↑ Biomass and seedling length ↑ NADPH metabolism ↑ Redox homeostasis ↓ Cd accumulation in roots and shoots	↑ G6PDH and antioxidant gene expression ↑ Activated APX, GR, and MDHAR ↓ NOX activity Balanced AsA‐GSH pools	Sakouhi et al. (2023)
Cabbage (copper)	0.5 and 1 mM CuSO_4_; 8 d	1, 10, and 100 μM; 3 d (Hydro)	Petri dish	↑ Membrane and DNA integrity ↑ Seedling growth at 1–10 μM 1–10 μM reversed copper toxicity High MLT (100 μM) was toxic	↑ Tolerance only at low dose ↓ Blocked copper‐induced lipid peroxidation ↓ Oxidative stress Maintained cell division	Posmyk et al. (2008)
Wheat (chromium)	100 μM Cr (Ⅵ) as K_2_Cr_2_O_7_; 7 d	100 μM; 12 h (Hydro)	Petri dish	↑ Germination and chromium accumulation ↑ Reserve mobilization ↑ Antioxidant levels	↑ SOD, CAT, APX, and POD activities ↑ Sugar/amino acid content ↑ Osmotic regulation ↓ NOX genes (*TaRbohD*/*F*)	Lei et al. (2021)
Tomato (cadmium)	100 μM CdCl_2_; 7 d	10, 50, 100, 150, and 200 μM; 24 h (Hydro)	Petri dish	↑ Germination, vigor index, and root length at 100–150 μM ↑ Synergistic action is stronger than on its own ↓ Cadmium and MDA levels	↑ SOD, CAT, and POD activities ↑ Radicle length and weight ↓ ROS via synergy	Lv et al. (2023)
Buckwheat (cadmium)	0.5 and 1.0 ppm CdCl_2_; 5 and 14 d	50 μM; 3 h (Hydro)	Petri dish	↑ Biomass ↑ Osmolyte balance ↑ Mineral uptake ↓ Growth, water potential, and lignin	↑ Osmotic adjustment ↑ Nutrient uptake ↓ Lignification and phenolics ↓ Oxidative damage	Colak (2025)
Rice (aluminum)	150 μM AlCl_3_; 6 d	10, 50, and 100 μM; 8 h (Hydro)	Petri dish	↑ Germination rescued at 10–100 μM ↑ Root elongation and biomass ↑ Citric acid secretion ↓ ROS and MDA levels	↑ SOD, CAT, AsA, and GSH activities ↑ α‐amylase upregulated ↓ ABA downregulated ↓ Cell wall‐bound aluminum ↓ Hemicellulose and pectin	Jiang et al. (2025)
*Plantago ovata* (lead)	50, 100, 200, and 500 μM Pb(NO_3_)_2_; 10 d	25 and 50 μM; overnight (Hydro)	Petri dish	↑ Biomass and photosynthesis at 25–50 μM ↑ Antioxidant enzyme activities ↓ Lead and stress markers (H_2_O_2_ and MDA) ↓ Ca^2+^ regulation limited lead uptake	↑ POD, APX, and SOD activities ↑ Strengthened antioxidant network ↓ ROS and lipid peroxidation ↓ Blocked lead via Ca^2+^ channel modulation	Chakraborty and Raychaudhuri (2024)
*Plantago ovata* (lead)	50, 100, 200, and 500 μM Pb(NO_3_)_2_; 10 d	50 μM; 12 h (Hydro)	n/a	↑ Flavonoids and anthocyanins ↑ Growth and hormonal balance ↓ Lead uptake and ROS damage ↓ Lead transporter gene	↑ Expressions of *PoPAL*, *PoPPO*, *PoMYB*, and *PoCOMT* ↑ IAA level ↑ Antioxidative status ↓ ABA accumulation and lead transporter (*PoMT2*)	Chakraborty and Raychaudhuri (2025)

Note: In some cases, multiple MLT doses were used in the experiment, and the best‐performing MLT doses are mentioned in the “Key findings” columns. Upward green arrows (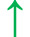) indicate increased, accumulation, or upregulated mechanisms and the downward red arrows (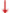) indicate decreased, suppressed, or downregulated mechanisms. “n/a” means that this information is not available in the article.

Abbreviations: ABA, abscisic acid; APX, ascorbate peroxidase; AsA, ascorbate; CAT, catalase; Ca^2+^, calcium ions; Chl, chlorophyll; d, days; EL, electrolyte leakage; FC, field capacity; GA, gibberellin; GSH, glutathione; GR, glutathione reductase; H_2_O_2_, hydrogen peroxide; hydropriming, hydro; h, hour; IAA, indole acetic acid; MDA, malondialdehyde; MLT, melatonin; MSP, melatonin seed priming; MDHAR, monodehydroascorbate reductase; POD, peroxidase; K^+^, potassium ions; K^+^, potassium ions; RWC, relative water content; ROS, reactive oxygen species; SOD, superoxide dismutase; Na^+^, sodium ions.

### Drought stress

Drought or water deficit stress severely impairs seed germination, root development, photosynthesis, and overall plant growth due to cellular dehydration, oxidative damage, and hormonal imbalances (Toulotte et al., 2022; Cooper and Messina, 2023; He et al., 2024; Sato et al., 2024; Raza et al., 2026b). During the early seedling developmental stage, MSP establishes early redox balance, osmoprotection, and hormonal homeostasis that pre‐condition plants for subsequent water deficit. These responses align with general MSP‐induced redox and osmotic adjustments, but display stress‐specific modulation in timing and intensity (Table 1).

A major benefit of MSP is improved drought tolerance, as evidenced by measurable changes in below‐ and above‐ground traits, particularly root system architecture, hydraulic conductivity, and biomass maintenance. This benefit refers to the degree (effect size) of physiological improvement observed across multiple studies rather than a single mechanistic effect, which has been consistently reported under both drought‐ and PEG‐induced osmotic stress. For example, MLT priming significantly improved root elongation and hydraulic conductance by upregulating the expression of aquaporin genes, ultimately enhancing root length, water transport, and biomass in wheat under combined drought and salinity stresses (Fu et al., 2024). Similar trends were observed in maize (Muhammad et al., 2023), rice (Tyagi et al., 2025), peanut (de Camargo Santos et al., 2024), triticale (Guo et al., 2022), and sunflower (Kumari et al., 2026), where MSP preserved chloroplast integrity and increased seedling and root length, biomass of roots and shoots, and stomatal conductance. These responses suggest improved water use efficiency (WUE) and enhanced carbon assimilation under stress. However, the impact of MSP on WUE is context‐dependent, since WUE shows the balance between carbon gain and water loss. Excessive stimulation of stomatal conductance without corresponding increases in root or photosynthetic activity could reduce WUE. On the contrary, coordinated adjustments across roots, leaves, and chloroplasts lead to a net gain in WUE and drought tolerance (Li et al., 2017; Wang et al., 2022).

A core mechanism by which MSP enhances drought tolerance is the potentiation of cellular defense and repair systems. MSP consistently activates a network of antioxidant enzymes, including superoxide dismutase (SOD), peroxidase (POD), and catalase (CAT), across diverse species, including maize (Muhammad et al., 2023), wheat (Shaheen et al., 2024), rapeseed (Khan et al., 2020), peanut (de Camargo Santos et al., 2024), pearl millet (Awan et al., 2024), and safflower (Heshmati et al., 2021). This enzymatic upregulation leads to effective ROS scavenging, which minimizes oxidative damage, as demonstrated by reductions in malondialdehyde (MDA), hydrogen peroxide (H_2_O_2_), and electrolyte leakage. These responses help maintain membrane integrity and cellular homeostasis under water‐limited conditions (Mittler et al., 2022; Wang et al., 2024a).

Concurrently, MSP promotes the accumulation of osmoprotectants and metabolic stabilizers. For instance, proline, glycine betaine, total soluble sugars, and amino acids were consistently increased in primed seedlings, which thus contributed to osmotic adjustment and cellular homeostasis under drought in different crops, for example, pearl millet (Awan et al., 2024), maize (Muhammad et al., 2023), and wheat (Shaheen et al., 2024). These accumulations build a foundational layer of MSP‐driven stress protection, while also contributing to cellular turgor maintenance and stress signaling, especially during seedling establishment.

Notably, MSP effects are dose‐dependent and genotype‐specific, which demand watchful optimization for practical application, as clearly demonstrated across 10 diverse maize genotypes where MSP (200 μM) was superior to GA_3_ priming (Kumari et al., 2026). Moreover, 2 mg L^−1^ MLT was most effective in wheat (Shaheen et al., 2024), 20 μM in triticale (Guo et al., 2022; Kolupaev et al., 2024) and rye (Kolupaev et al., 2024), 50 μM in peanut (de Camargo Santos et al., 2024), 120 ppm in rice (Tyagi et al., 2025), and 200 μM in sorghum (García‐Cánovas et al., 2024), which shows that responses vary with crop species, cultivars, and stress severity. Moreover, primed sorghum, rice, and barley seeds showed improved germination and seedling vigor even in aged seeds, which highlights that MSP may alleviate seed deterioration and extend seed longevity under drought (García‐Cánovas et al., 2024).

In addition to cellular and biochemical effects, MSP has also translated into measurable agronomic benefits, including improved seedling establishment, increased biomass accumulation (in both shoots and roots), higher yield components, and better seed quality under drought and open‐field conditions in crops like wheat and rapeseed (Khan et al., 2020; Shaheen et al., 2024). This attractive scalability highlights its practical importance for semi‐arid and rainfed agriculture.

### Salinity stress

Salinity stress imposes a combination of osmotic and ionic constraints on plant performance, that is, osmotic imbalance and ion toxicity. This results in impaired seed germination, reduced seedling vigor, disrupted nutrient uptake, and excessive ROS accumulation (Liang et al., 2024; Tahjib‐Ul‐Arif et al., 2025; Yuan et al., 2025; Raza et al., 2025c; Wang et al., 2026a). Emerging research supports the role of MSP in mitigating these detrimental effects through multifaceted physio‐biochemical and molecular mechanisms (see Table 1 for detailed analysis). Across a wide range of crops, including cereals (wheat, maize, sweet corn, sorghum, and rice), legumes (soybean and common bean), halophytes, and desert plants, MSP significantly enhances germination rates, root and shoot growth, photosynthetic efficiency, and stress tolerance thresholds (as detailed in Table 1). These benefits largely arise from coordinated regulation of redox balance, hormonal signaling, and metabolic adjustment.

As observed under drought stress, the potentiation of antioxidant defenses is a key mechanism by which MSP mitigates salinity. Priming with MLT enhances the activity of SOD, CAT, POD, and APX, which effectively mitigates oxidative damage, as indicated by reduced levels of MDA and H_2_O_2_, as noticed in tartary buckwheat (Zhu et al., 2025b), *Suaeda corniculata* (Zhang et al., 2024a), and soybean (Awan et al., 2023). However, salinity imposes the additional load of “ion toxicity”, and here, MSP serves a unique and critical function. It actively re‐establishes “ionic homeostasis” by limiting the accumulation of toxic sodium ions (Na^+^) while improving the uptake of essential ions such as potassium (K^+^), calcium (Ca^2+^), and zinc (Zn^2+^). This rebalancing is supported by improved K^+^/Na^+^ and Ca^2+^/Na^+^ ratios in crops such as sorghum (Kiremit et al., 2024), common bean (Alinia et al., 2021), and sweet corn (Sezer et al., 2021), and *Salvia miltiorrhiza*, where 20 μM MSP improved the K^+^/Na^+^ ratio, elevated GA_3_, and downregulated ABA biosynthesis genes (Li et al., 2026). These changes are vital to prevent cytosolic ion toxicity and maintain metabolic function. Importantly, this restoration of ionic homeostasis and nutrient uptake affects the nutritional composition of edible plant organs. Thus, MSP in saline conditions integrates the common antioxidant response with a specific ion‐homeostatic mechanism to confer tolerance.

Though most MSP studies have focused on early growth traits (i.e., roots, shoots, or leaves), the observed upsurges in K^+^, Ca^2+^, Zn^2+^, manganese, and copper uptake (Sezer et al., 2021; Kiremit et al., 2024) suggest a potential, yet underexplored, role for MSP in safeguarding the nutritional quality of food and feed under salinity stress. Therefore, future efforts should link MSP‐driven ionome shifts to seed or fruit nutritional profiles that would add a vital food security dimension to this approach. In short, this dual action (ROS detoxification and ionic balance restoration) reflects MSP's essential role as a cellular stabilizer under salinity.

According to soaking‐based MSP, novel MLT delivery systems have been developed to achieve sustained protection. For example, Athanasiou et al. (2025) engineered calcium alginate‐based hydrogel biopolymers (HYDR‐MLT) that gradually release MLT during germination, thus improving germination indicators, root elongation, and antioxidant status in tomato under salinity stress. These green hydrogel coatings are an innovative next‐generation seed treatment method that integrates hormone‐mediated priming with environmentally friendly materials to mitigate salinity‐induced oxidative/osmotic stress.

In wheat, MSP (500 μM) not only overturned reductions in germination, amylase activity, and antioxidant enzyme levels under salinity but also restored photosynthetic capacity (Fv/Fm and ΦPSII), Chl content, sucrose, and the relative water content (RWC). Furthermore, it suppressed salinity‐induced upsurges in MDA, H_2_O_2_, superoxide (O_2_^•−^), and Na^+^ levels (Ismaeil et al., 2024). These enhancements emphasize MSP's integrative function in improving metabolic vigor, photosynthetic integrity, and detoxification under high salinity, as well as hormonal rebalancing in the medicinal herb *Salvia miltiorrhiza* (Li et al., 2026).

In maize (Ismaeil et al., 2025) and sorghum (Kiremit et al., 2024), MSP reversed salinity‐induced reductions in biomass, Chl, and soluble sugar content, while alleviating RWC and carotenoid levels. Specifically, maize seedlings primed with 1,000 μM MLT showed enhanced growth, RWC, antioxidant activities, and photosynthetic pigment concentrations, while Na^+^, abscisic acids (ABA), ROS, and lipid peroxidation were substantially repressed (Ismaeil et al., 2025). In two halophyte species, including *Zygophyllum simplex* and *Portulaca oleracea*, even low MLT concentrations (5 and 100 μM) were effective in alleviating seed dormancy, improving germination under salinity stress, and enhancing seedling photosynthetic pigment accumulation (Hussain et al., 2024). Higher doses (e.g., 250–500 μM) were generally less effective or inhibitory. Similarly, in soybean (Awan et al., 2023) and cotton (Zhang et al., 2021b), MLT doses of 20–100 μM significantly enhanced germination potential, shoot/root biomass, and radical elongation under combined stresses, mainly by boosting antioxidant systems and mitigating oxidative stress. These outcomes indicate that MLT regulates water status, photosynthesis, and hormonal homeostasis under salinity stress.

Another MLT‐mediated stress tolerance mechanism is hormonal and genetic reprogramming. In cotton, MSP modulated phytohormone signaling pathways (including ABA, ethylene, and auxin) and upregulated genes associated with ROS detoxification and stress signaling. In contrast, it downregulates photosynthesis‐related genes to conserve energy under stress (Zhang et al., 2021b). In common bean, the synergistic effect of MLT and *Rhizobium* inoculation not only boosted photosynthetic pigment retention and antioxidant system but also enhanced seed yield, which suggests a novel strategy for bio‐enhanced stress mitigation (Alinia et al., 2022b). This regulatory complexity indicates that MLT not only primes antioxidant machinery but may also rewire hormonal circuits and root–microbe interactions, which can serve as a multi‐layered defense against soil salinity. Similar synergistic benefits were observed when MSP was combined with SA in two sunflower hybrids, where the combination reduced electrolyte leakage, H_2_O_2_, and MDA while improving root/shoot fresh weight, with stronger responses in the more sensitive hybrid “ORISUN‐741” (Zia et al., 2026).

The role of MLT in osmoregulation is equally dynamic under salinity. For instance, in *Sophora alopecuroides* (Zhang, 2025) and desert species such as *Sarcozygium xanthoxylon*, *Nitraria tangutorum*, and *Ammopiptanthus mongolicus*, low MSP levels optimized osmolyte (proline) accumulation and antioxidant enzyme activities, which enable these plants to flourish in saline–alkaline soils (Zhang et al., 2024b). However, extremely high MLT concentrations (e.g., 250–300 μM) were irregularly inhibitory, and this outcome emphasized the importance of dose optimization for maximum benefit (Zhang et al., 2024a; 2024b). These findings highlight the critical need for precision in MLT dosing to balance benefits and prevent overstimulation or phytotoxicity under stress.

### Extreme temperature stress

Extreme temperature stress (low temperature, chilling (0°C–15°C) or freezing (< 0°C), and high temperature (> 25°C)) refers to variations in the optimal range for germination and early seedling growth, and depends on the plant species (Castroverde and Dina, 2021; Raza et al., 2022; Sato et al., 2024; Kang et al., 2025; Zeng et al., 2026). These extremes are ever more frequent under current changing climates and are predicted to intensify (https://www.ipcc.ch/sr15/), and will continue to pose significant threats to seed vigor, establishment, and yield stability. Particularly, these extreme events disrupt plant metabolism, delay, or impair germination, and weaken seedling establishment, often by inducing oxidative damage, metabolic imbalances, and membrane instability (Castroverde and Dina, 2021; Raza et al., 2022; Kerbler and Wigge, 2023). Despite comparatively limited data compared to drought or salinity, recent studies suggest that MSP may be a fruitful strategy for coping with temperature stress during early developmental stages (see Table 1 for detailed insights).

Under cold conditions, MSP constantly boosts germination and seedling vigor across diverse crops, including waxy maize (Cao et al., 2019), tomato (Park et al., 2025), wheat (Zhang et al., 2021a), and pepper (Korkmaz et al., 2017). These benefits are largely driven by enhanced antioxidant systems (SOD, CAT, POD, and APX), reduced MDA levels, and improved membrane stability, which collectively reduce oxidative damage. Notably, MSP also accelerates the expression of cold‐responsive genes such as *C‐repeat binding factors* (C*BFs*), thus boosting cold acclimation in tomato (Park et al., 2025). The *OsbZIP83‐OsCOMT15* module confers MLT‐ameliorated cold tolerance (Li et al., 2025), and MSP alleviates cold stress via *OsABI5*‐mediated signals during seed germination in rice (Li et al., 2021b). Studies in onion and leek seeds (Hancı et al., 2019) and mung bean (Szafrańska et al., 2014) further emphasize MSP's ability to preserve cellular homeostasis and reduce electrolyte leakage under chilling and re‐warming cycles.

In maize (Cao et al., 2019) and wheat (Zhang et al., 2021a), MSP promoted starch metabolism and increased soluble carbohydrate accumulation under cold stress, which suggests MSP's role in energy balance and osmoprotection. Proteomic‐driven investigations also indicate that MSP drives significant shifts in seed protein profiles under cold conditions, thus potentially priming the maize seeds for enhanced metabolic readiness and stress perception (Kołodziejczyk et al., 2016).

Under heat stress, MSP confers tolerance by safeguarding photosynthetic pigments, minimizing ROS generation, and enhancing enzymatic antioxidant activities. For instance, MLT notably reduced heat‐induced oxidative stress markers in soybean (Awan et al., 2023) and in cereals like wheat and rye (Kolupaev et al., 2023), and also improved seedling vigor and the growth of both shoots and roots. These changes were associated with increased CAT activity, maintained or enhanced POD activity, and the accumulation of osmoprotectants, for example, soluble sugars.

Importantly, while the dose response is often species‐dependent, low to moderate MLT concentrations (5–100 μM) appear to be steadily effective, particularly when combined with hydro‐ or drum‐priming techniques. For instance, in soybean (Awan et al., 2023), pepper (Korkmaz et al., 2017), mung bean (Szafrańska et al., 2013, 2014), and wheat/rye (Zhang et al., 2021a; Kolupaev et al., 2023), 20–100 μM MLT improved germination and seedling vigor under both heat and cold. Similarly, in tomato, drum priming with MLT improved emergence and early cold tolerance (Park et al., 2025), while in waxy maize, 50–100 μM MLT enhanced antioxidant enzyme activity and radicle growth under chilling (Cao et al., 2019).

### Heavy metal toxicity

Heavy metal contamination creates a significant challenge for seed germination and seedling establishment by triggering oxidative stress, disrupting membrane integrity, altering nutrient homeostasis, and interfering with hormonal and metabolic balance (Tang et al., 2023b; Charagh et al., 2024). However, it is essential to note that the phytotoxic effects are highly dose‐dependent. For instance, elevated concentrations are detrimental; nevertheless, a recent review specifies that low metal levels, for example, cadmium (Cd), can induce hormetic effects, which can lead to neutral or even enhanced seed germination and seedling vigor in some species (Carvalho et al., 2023). This dual nature of Cd response highlights that while excessive exposure is toxic (Qiao et al., 2019; Charagh et al., 2025), mild Cd doses can act as a priming stimulus, which can activate several physio‐biochemical mechanisms (Carvalho et al., 2023). MSP research on metal toxicity remains limited, and existing evidence strongly supports the potential of MSP to mitigate metal stress through multilayered protective mechanisms (Table 1).

The literature shows that MSP can efficiently protect seeds against the toxic effects of metals such as Cd, aluminum (Al), chromium (Cr), copper (Cu), and lead (Pb), by modulating antioxidant systems, limiting metal uptake, and improving early‐stage physiological vigor. For instance, in wheat under Cr toxicity (Lei et al., 2021) and chickpea under Cd toxicity (Sakouhi et al., 2023), MSP increases seedling growth and biomass by boosting reserve mobilization and improving sugar and amino acid availability, but downregulating metal‐induced ROS‐producing enzymes such as NADPH oxidases. This is often accompanied by the activation of antioxidant enzyme systems (SOD, CAT, POD, and APX), which ensured redox homeostasis during early development, as noticed in Al‐stressed rice (Jiang et al., 2025), Cu‐stressed rice (Song et al., 2021), and Pb‐stressed *Plantago ovata* (Chakraborty and Raychaudhuri, 2024).

MSP also influences ion transport and metal sequestration mechanisms to stop metal translocation into sensitive tissues. For example, MSP in rice enhanced organic acid secretion, particularly citrate, which reduces Al accumulation in root tissues and reduces toxicity symptoms (Jiang et al., 2025). Similarly, buckwheat (Colak, 2025) and *Plantago ovata* (Chakraborty and Raychaudhuri, 2024) seedlings showed decreased lignification and Pb (in *Plantago*) and Cd (in buckwheat) accumulation due to MLT‐mediated modulation of phenolic metabolism and Ca^2+^ channel activity.

Hormonal reprogramming is another crucial element of MSP‐mediated metal stress tolerance. MSP in *Plantago ovata* (Chakraborty and Raychaudhuri, 2025) and rice (Jiang et al., 2025) has been shown to suppress stress‐induced ABA accumulation while improving growth‐promoting IAA levels, which facilitate better root elongation and seedling emergence under Pb and AI toxicity. These hormonal shifts are maintained by increased expression of stress‐regulatory transcription factors (TFs; e.g., *PoMYB*) and secondary metabolite biosynthesis genes (*PoPAL*, *PoPPO*, and *PoCOMT*) in *Plantago ovata*, which suggests a role for MLT in activating both enzymatic and non‐enzymatic defense systems (Chakraborty and Raychaudhuri, 2025).

Interestingly, studies also emphasize the dose sensitivity of MSP under metal stress. For instance, low to moderate concentrations (10–100 μM) generally offer protective effects; on the other hand, excessive MLT can exacerbate metal toxicity, as observed in cabbage under Cu stress (Posmyk et al., 2008), which emphasizes the need for species‐ and stress‐specific optimization. Integrative priming strategies (e.g., combining MLT with other signaling molecules‐GABA) have shown synergistic benefits in tomato under Cd stress, which represent a new frontier of combinatorial biostimulant approaches (Lv et al., 2023).

### Nutrient deficiency

Nutrient deficiency stress remains one of the most overlooked constraints affecting seed germination, early seedling vigor, and crop productivity. Essential macronutrients (e.g., nitrogen, phosphorus, potassium, or magnesium) and micronutrients (e.g., iron or nickel) are important for redox regulation, Chl biosynthesis, enzyme function, and energy metabolism. Deficiencies in these nutrients often result in disrupted photosynthetic capacity, altered root architecture, and oxidative stress during early plant development (Tahjib‐Ul‐Arif et al., 2025; Wang et al., 2025). Although most MLT studies under nutrient deficiency involve foliar or hydroponic treatments, direct evidence for MSP remains very limited and should be a new research target. Recent investigations suggest that seed‐based delivery could pre‐condition plants for improved nutrient utilization efficiency (Bouzidi and Krouma, 2025; Tahjib‐Ul‐Arif et al., 2025). However, it is important to note that MSP is usually applied to seeds with ample internal reserves, and its special effects during germination may depend less on external nutrient availability and more on internal metabolic reprogramming. Nevertheless, early MLT signaling triggered by MSP may “pre‐tune” nutrient‐sensing and antioxidant pathways, which could support successive nutrient uptake and allocation once seedlings transition to heterotrophic growth.

To date, only one study has directly tested MSP under magnesium (Mg) deficiency stress (Bouzidi and Krouma, 2025). This new MSP study demonstrated that MLT‐primed common bean seeds showed improved tolerance to Mg deficiency through enhanced Chl retention, better PSII photochemical efficiency, and superior redox regulation. Notably, genotype‐dependent responses indicated that MSP promotes more effective energy management in the photosynthetic machinery, particularly in tolerant cultivars (Bouzidi and Krouma, 2025). These findings indicate the untapped potential of MSP to enhance nutrient‐stress tolerance, especially in crops adapted to low‐input systems.

### Waterlogging/Flooding stress

Waterlogging and flooding stress severely disrupt seed germination and seedling establishment by inducing hypoxia, triggering hormonal imbalances, and overproducing ROS, all of which suppress metabolic activity and root functionality (Toulotte et al., 2022; Daniel and Hartman, 2024; Renziehausen et al., 2025). Nevertheless, research on MSP under waterlogging stress remains extremely limited, with only a handful of studies available, as discussed below.

In wheat, maize, alfalfa, and soybean, transcriptome‐level evidence indicated that MSP significantly remodels hormonal signaling and redox metabolism under flooding stress, which boosted germination and early growth even under oxygen‐deprived conditions. Specifically, MSP modulates ABA–gibberellin (GA) antagonism by upregulating GA biosynthesis genes (e.g., *GA20ox*), promoting ABA catabolism, and downregulating ABA biosynthetic genes, which effectively relieve dormancy and stimulate seed metabolism (Luo et al., 2024). Simultaneously, MSP boosts ROS‐scavenging enzymes, which protect cells during hypoxic episodes in rice plants (Zeng et al., 2022a).

In maize, a dual priming strategy using MLT and KNO_3_ not only promoted growth under waterlogging stress but also improved photosynthetic rates, decreased the activities of pyruvate decarboxylase and alcohol dehydrogenase (key fermentation enzymes), and reduced oxidative damage markers such as H_2_O_2_ and MDA (Ahmad et al., 2022). These changes highlight a metabolic shift from fermentative survival to aerobic‐like energy efficiency, which is made possible by pre‐programming seeds via MLT priming.

Seed priming effects appear to be genotype‐dependent. For instance, in direct‐seeded rice, seed priming with a mix of ABA and MLT enhanced germination and antioxidant enzyme activities even under the combined stress of cold and submergence, with mainly notable improvements in sensitive cultivars (Ma et al., 2025). This highlights the potential of MSP not just for flooding alone but also for complex stress combinations, where temperature and oxygen deprivation co‐occur. Still, the mechanistic understanding remains incomplete, particularly regarding its interactions with hypoxia‐inducible TFs (e.g., ERF‐VII), energy metabolism, and mitochondrial integrity during submergence.

### Combined abiotic stresses

Plants in natural environments rarely encounter a single stress in isolation; instead, combined stresses (e.g., drought + salinity, salinity + nutrient imbalance, heat + drought, etc.) often co‐occur in the field, which may intensify their negative impacts on growth and production (Zandalinas et al., 2024; Raza et al., 2025b). However, recent global analysis and multi‐factor experiments show that the number of concurrent environmental stresses has a compounding adverse effect on key ecosystem processes, including those in soils where seeds germinate and seedlings establish (Rillig et al., 2019, 2023). These studies highlight that soil processes, biodiversity, and productivity cannot be anticipated from single‐factor responses (Rillig et al., 2019, 2023), because of which there is an urgent need to assess plant responses under multi‐factorial stress combinations (*n* ≥ 2) (Zandalinas et al., 2024; Raza et al., 2025b).

Although foliar/exogenous MLT applications under combined stresses have been widely explored previously, MSP remains largely underexplored and requires systematic investigation to fully harness its potential in such setups. Combined drought–salinity stress severely reduces root hydraulic conductivity (Lpr); however, a recent study in wheat shows that MSP can partially restore Lpr by upregulating aquaporin genes and promoting root elongation (Fu et al., 2024). In addition, MSP enhanced antioxidant activities, stabilized osmotic balance, reduced MDA accumulation, and maintained higher K^+^ levels, which collectively supported seedling growth under combined stresses. Genotype‐dependent responses were also evident, which emphasized the need for tailored strategies (Fu et al., 2024).

Given that plants simultaneously face multiple climatic and edaphic constraints (Rillig et al., 2019; Zandalinas et al., 2024), integrating MSP‐driven investigations could be vital, as MSP pre‐activates a multifaceted defense portfolio. This strategy aligns with emerging global‐change biology concepts and will help test whether MSP can buffer plants against the accelerating cascade of interacting stresses that threaten ecosystem adaptation and food security. Therefore, we argue that future studies should expand beyond single‐stress ideas and test MSP under diverse stress combinations to leverage its practical potential for next‐generation seed technologies.

### Similarities and differences across stress types: A brief appraisal

Across stresses, MSP activates core mechanisms, that is, the potentiation of antioxidant enzyme systems, the accumulation of osmolytes, and the modulation of ABA–GA hormonal balance (Table 1). These conserved responses form a foundational layer of stress protection that is broadly effective. However, stress‐specific adaptations distinguish MSP's mode of action. MSP uniquely restores ionic homeostasis under salinity by limiting Na^+^ accumulation while enhancing K^+^, Ca^2+^, and Zn^2+^ uptake (Alinia et al., 2021; Kiremit et al., 2024). Under metal stress, MSP promotes metal sequestration by secreting organic acids and modifying the cell wall (Colak, 2025; Jiang et al., 2025). During drought, MSP enhances root hydraulic conductivity by upregulating aquaporin genes (Fu et al., 2024). MSP shifts metabolism from fermentative to aerobic pathways under waterlogging stress (Ahmad et al., 2022). Temperature stress responses are distinguished by the activation of *CBF* TFs and starch metabolism (Cao et al., 2019; Park et al., 2025). These stress‐specific mechanisms, covered by a common antioxidant system, explain MSP's versatility while highlighting the need for stress‐adapted protocols.

### A seed stage‐specific mechanistic model of MSP‐driven stress tolerance

**Figure 5:**
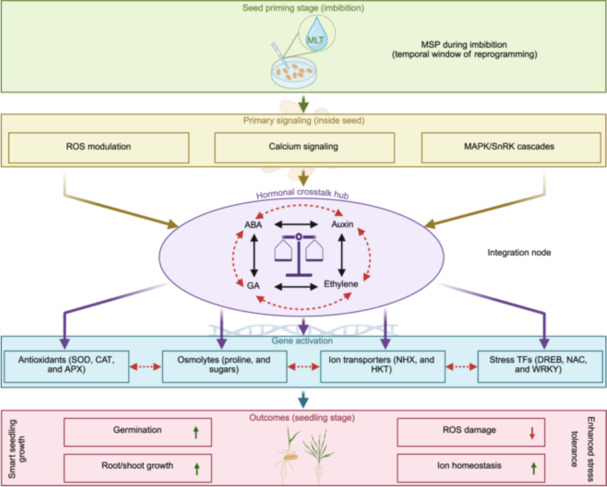
A seed‐stage‐specific mechanistic model of melatonin (MLT) seed priming‐driven stress tolerance MLT seed priming (MSP) functions during seed imbibition, during which exogenous melatonin establishes a primed state via controlled ROS, calcium, and MAPK/SnRK signaling. These signals converge on a hormonal crosstalk hub, rebalancing hormone levels to promote germination. Downstream activation of antioxidant genes, osmoprotectants, ion transporters, and stress TFs enhances seedling vigor, root/shoot growth, and stress tolerance. Solid arrows indicate direct MSP evidence and dashed red arrows show evidence inferred from non‐MSP studies (requiring validation). The model distinguishes MSP‐specific mechanisms from general melatonin biology and positions MSP as a seed‐stage trigger for stress tolerance. Upward green arrows (↑) indicate increased or upregulated mechanisms and downward red arrows (↓) indicate decreased or suppressed mechanisms. Created in https://BioRender.com. ABA, abscisic acid; APX, ascorbate peroxidase; CAT, catalase; GA, gibberellic acid; HKT, high‐affinity potassium transporter; NHX, sodium/hydrogen exchanger; ROS, reactive oxygen species; SOD, superoxide dismutase; TFs, and transcription factors.

Building upon the mechanisms summarized above, we propose a seed stage‐specific mechanistic model that discriminates MSP from general MLT‐mediated stress responses (Figure 5). In contrast to foliar applications, MSP operates within a narrow temporal window during seed imbibition, where exogenous MLT interacts with early hydration‐driven metabolic reactivation to establish a primed cellular state. This state is characterized by rapid modulation of redox homeostasis, including controlled ROS accumulation that acts as signaling cues rather than damage signals (Balmer et al., 2015; Sakouhi et al., 2023; Jiang et al., 2025).

At the signaling level, MSP operates within a core regulatory triangle involving ROS–hormone–calcium crosstalk. MSP constantly downregulates ABA biosynthesis genes (*NCED3* and *NCED5*) while upregulating GA biosynthesis genes (*GA20ox*, *SmKO*, and *SmKAO1*), tipping the balance toward germination‐promoting hormones (Zhang et al., 2021b; Luo et al., 2024; Li et al., 2026). This hormonal shift is mediated by MLT‐induced H_2_O_2_ accumulation, which activates ABA catabolism via *CYP707A* genes and Ca^2+^ signaling through *CAX3* (Li et al., 2021a). Concurrently, Ca^2+^ fluxes and MAPK/SnRK signaling cascades act as central integrators, transducing early redox signals into transcriptional reprogramming (Khan et al., 2020; Li et al., 2021a; Supriya et al., 2024).

Downstream, stress‐responsive TFs are activated in a stress‐specific manner. These collectively enhance antioxidant capacity, osmolyte accumulation, and ion homeostasis during early seedling establishment (Supriya et al., 2024; Chakraborty and Raychaudhuri, 2025; Park et al., 2025). For instance, MSP upregulates *CBFs*, coordinating *COR* gene expression for cold tolerance (Park et al., 2025), and ABA‐independent MAPK–SnRK2–SnRK1 signaling triggers autophagy‐related genes (*ATG8* and *RAPTOR1*) under drought conditions (Supriya et al., 2024). Under salinity, MSP modulates ion transporters (*HKT1*, *SOS1*, and *NHX1*) for Na^+^ exclusion alongside antioxidant genes (*CAT*, *SOD*, and *APX*) (Zhang et al., 2021b; Alinia et al., 2022b). For heavy metals, MSP induces vacuolar sequestration via *HMA3* and *NRAMP5*, and organic acid biosynthesis via *ALMT* and *MATE* for metal chelation (Colak, 2025; Jiang et al., 2025). This multi‐layered network, which includes an upstream hormonal–ROS hub, a mid‐level TFs' cascade, and downstream effector genes, provides insights into how the same MLT signal yields stress‐specific outputs.

Nevertheless, the persistence of these responses to later developmental stages is frequently observed. However, the extent to which this reflects true epigenetic stress memory versus transient metabolic priming remains unresolved. This points to a working hypothesis rather than a confirmed mechanism (Figures 1, 5). This seed‐stage‐specific model distinguishes MSP from general exogenous MLT effects and highlights the need to validate potential epigenetic memory (Figure 1). This model also provides a conceptual basis for integrating MSP with omics‐driven validation and predictive modeling approaches in future studies.

## A CRITICAL ASSESSMENT OF MSP: LIMITATIONS, INCONSISTENCIES, AND KNOWLEDGE GAPS

In addition to MSP‐driven stress tolerance, a closer examination highlights substantial context dependency, methodological limitations, and translational gaps. Hence, we provide a systematic appraisal of these issues.

### Dose‐dependent and hormetic responses: A brief systematic analysis

**Figure 6:**
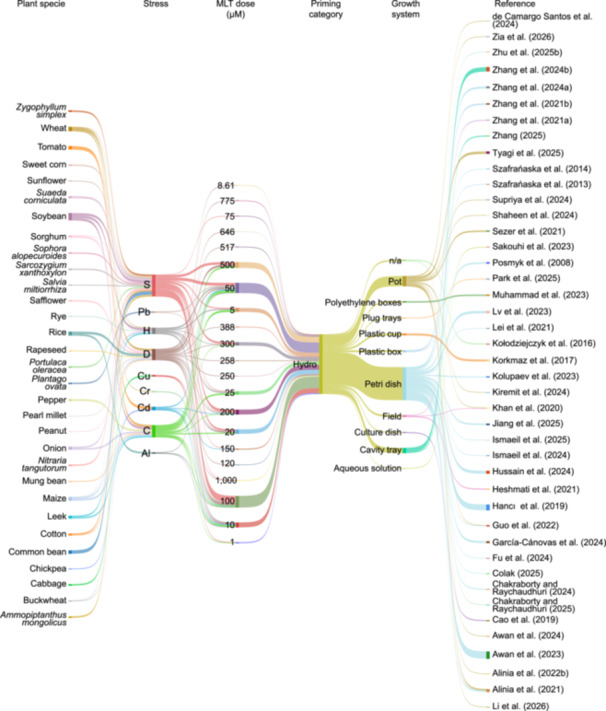
A Sankey map visualizing the dose–response landscape of melatonin seed priming (MSP) The diagram classifies effective and inhibitory MSP concentrations based on studies compiled in Table 1. For a comparative analysis across studies, we converted some of the dose values into a common unit (μM). This diagram was created using an online Bioinformatics platform (https://www.bioinformatics.com.cn/) (Tang et al., 2023a) and finalized with Adobe Illustrator. AI, aluminum; Cd, cadmium; Cr, chromium; Cu, copper; C, cold; D, drought; H, heat; Hydro, hydropriming; Pb, lead; MLT, melatonin; Ni; and S, salinity. n/a means not available.

MSP responses follow a classic hormetic pattern, with benefits at low to moderate doses and diminished/inhibitory effects at high doses. A systematic analysis of reviewed studies (Figure 6; Table 1) suggests three key patterns. First, effective doses span a wide range (≤ 10 μM to 1,000 μM), but medium doses (51–200 μM) are most frequently associated with positive outcomes across stress types and species. Second, negative or inhibitory effects largely initiate from high (> 200 μM) and very high (> 500 μM) doses, as illustrated by intensified copper toxicity in cabbage at 100 μM (Posmyk et al., 2008) or loss of efficacy in *S. corniculata* > 50 μM under salinity (Zhang et al., 2024a). Third, very low doses (≤ 10 μM) remain underexplored for most crops, while a recent meta‐analytical suggested the strong average benefits at these levels (Agathokleous et al., 2021). This calls for more MSP‐driven studies using very low level MLT across stresses and species. Further arguments related to dose response are discussed in the above stress‐specific sections.

Treatment duration and seed characteristics add further complexity. Both inadequate and excessive soaking times can reduce efficacy, while seed size, coat permeability, and baseline vigor modulate uptake. Notably, this wide therapeutic window is highly context‐dependent, for example, doses effective in one species–stress combination may be suboptimal or even inhibitory in another (as discussed earlier in each stress section). Briefly, MSP with 20 μM MLT effectively alleviated drought stress in triticale (Guo et al., 2022), whereas rapeseed required 500 μM for equivalent benefits (Khan et al., 2020). This highlights the limited transferability of current protocols. These findings confirm that generic statements about dose optimization are insufficient and the notion that “more is better” is essentially flawed for MSP. Thus, the field must move forward toward systematic, multi‐factorial dose–response matrices that account for species, stress type, severity, and duration, particularly under combined stress scenarios, which remain almost entirely unexplored despite their field relevance.

### Genotype‐, species‐, and stress intensity‐dependent variability

Genetic background, species, and stress intensity strongly modulate MSP efficacy. Differential responses occur even within species, for instance, greater benefits in common bean cultivar “Coco Nain” versus “Coco Blanc” under Mg deficiency (Bouzidi and Krouma, 2025), or more consistent recovery across 10 maize genotypes under drought with 200 μM MSP compared to GA_3_ priming (Kumari et al., 2026). Similar genotype‐specific responses were observed in wheat under combined drought–salinity stress (Fu et al., 2024) and in desert plants, where the optimal synergistic dose of MLT and IAA varied among species (Zhang et al., 2024b).

As quantified in Table 1, drought and salinity dominate the literature (~65% of studies), with MSP indicating high success rates (> 80%) under moderate stress intensities (e.g., ≤ 100 mM NaCl or 35%–40% field capacity). However, efficacy often declines under severe stress conditions, where cellular damage exceeds the priming‐induced protective capacity. This suggests that MSP is not universally effective across stress gradients. Efficacy is also reduced in genotypes with low basal antioxidant capacity or MLT responsiveness (Fu et al., 2024; Hussain et al., 2024; Ismaeil et al., 2025). Therefore, protocols optimized for one variety cannot be reliably extrapolated to another without validation.

Trait endpoints also vary in agronomic relevance. Most studies focus on early germination and biochemical markers (e.g., proline, MDA, and antioxidant enzymes), and only a few report yield elements or field performance (as detailed in the next section). This creates a critical gap between physiological responses and agronomic outcomes, and limits the predictive interpretation of MSP performance under substantial cropping systems. Building on the stress‐specific patterns, these findings highlight the need for studies on genotype × environment × dose interaction to move forward from generic claims of enhanced stress tolerance.

### Translational and practical bottlenecks: appraisal of post‐priming seed stability, and the field validation paradox

Two major practical limitations constrain MSP exploitation, including post‐priming seed stability and limited field validation. Priming can reduce desiccation tolerance, leading to continuing loss of vigor during storage and drying. For example, a recent study on tartary buckwheat demonstrated that the MSP benefits were eroded over prolonged drying and storage, with primed seeds losing vigor over time (Zhu et al., 2025b). This time‐dependent loss of efficacy, also highlighted by meta‐analytical data (Agathokleous et al., 2021), defines a critical post‐priming window for sowing. This creates a narrow safe sowing window that complicates commercial seed handling and distribution.

Compounding this issue is the field validation paradox. The majority of MSP studies are conducted in Petri dishes, growth chambers, or greenhouses, with fewer than 5% including multi‐location or multi‐year field trials that evaluate yield or yield stability (Abd El‐Ghany and Attia, 2020; Khan et al., 2020; Heshmati et al., 2021). Hence, it should be noted that controlled‐environment gains in seedling vigor or biochemical markers do not necessarily translate to field emergence or productivity under variable, multifactorial stresses.

This translational gap extends to critical implementation factors, including economic feasibility, regulatory considerations (e.g., MLT residues), and compatibility with existing seed‐industry practices (e.g., fungicide treatments, film coating, and pelleting). However, all of these remain largely untested. Without such data, the characterization of MSP as a scalable, sustainable seed‐based solution remains aspirational rather than evidence‐based.

### Conflation of MSP with foliar melatonin effects

A significant conceptual limitation in the current literature is the frequent conflation of mechanisms derived from seed‐priming studies with those inferred from foliar or hydroponic MLT applications. While both application methods may converge on similar pathways, the developmental contexts are fundamentally different. MSP acts during the critical early establishment stage, guiding early imbibition, reserve mobilization, and the establishment of primary meristems (Rajora et al., 2022).

However, direct molecular outcomes for MSP‐specific signaling remain limited, with many proposed mechanisms concluded from vegetative‐stage studies. This conceptual overlap can obscure the discovery of seed‐stage‐specific regulatory networks and lead to overly general mechanistic interpretations. In this context, we proposed a seed‐stage‐specific mechanistic model addressing this gap (Figure 5).

### Experimental rigor, comparative context, and agronomic relevance

Many MSP studies under‐report critical seed‐lot characteristics (baseline germination capacity, vigor, storage history, and health status), despite evidence that low‐vigor or aged seeds often show stronger priming responses (García‐Cánovas et al., 2024; Kolupaev et al., 2024). Inadequate replication, randomization, or statistical detail further limits generalizability. Moreover, direct side‐by‐side comparisons with other priming strategies (e.g., osmopriming, hormopriming, or microbial co‐application) are scarce, which makes it difficult to establish MSP's relative advantages, consistency, or additive value (Alinia et al., 2022b; Kumari et al., 2026).

Equally importantly, most studies rely on early‐stage laboratory endpoints, whereas hierarchical validation (e.g., from germination to field emergence, biomass accumulation, and ultimately yield stability) is rarely executed. This disconnect weakens agronomic relevance and limits the ability to position MSP within the broader priming toolkit.

These issues highlight the need for higher experimental standards and hierarchical trait assessment. Therefore, we argue that future studies should transparently report seed‐lot metadata, prioritize agronomically relevant endpoints, and conduct systematic comparisons with existing seed technologies. We anticipate that such advances can help harness the true benefit of MSP over conventional priming.

## SYNERGISTIC APPROACHES WITH MELATONIN SEED PRIMING

Though MSP alone has shown considerable potential to mitigate various abiotic stresses, combinatorial approaches hold great potential to boost stress tolerance and broaden applicability across species and environments. Given MLT's multifunctionality, its co‐application with other priming agents, nanomaterials, or biological inputs may represent a layered, systemic approach for stress adaptation.

### Combination with other priming agents

MSP significantly enhances germination and early seedling vigor under stress, but its integration with other priming agents, for example, phytohormones, osmoprotectants, and metabolic intermediates, unchecks synergistic effects that outperform individual treatments. For example, a recent study discovered that co‐priming with MLT and plant growth regulators like ABA, SA, GA_3_, and auxins activates multi‐layered defenses, including enhanced antioxidant enzyme activities, reduced lipid peroxidation, and improved seedling establishment under cold and submergence conditions in rice (Ma et al., 2025) and drought stress in canola (Rafique et al., 2024). These combinations mitigate oxidative injury and enhance endogenous MLT and SA levels, which indicate a priming‐induced feedback amplification loop that stabilizes stress signaling networks (Rafique et al., 2024; Ma et al., 2025). In sunflowers, MLT + SA co‐priming further enhanced morphological traits and antioxidant activities under salinity (Zia et al., 2026). Likewise, a direct comparison showed that MSP (200 μM) was superior to GA_3_ priming for restoring germination, osmolyte accumulation, and antioxidant status under drought stress across multiple maize genotypes (Kumari et al., 2026).

MSP‐driven synergy mobilizes antioxidant enzymes (SOD, CAT, and APX), osmolytes (proline and sugars), and nutrient transporters, which jointly reduce oxidative damage and maintain membrane integrity across diverse species under various stresses, for example, Cd in tomato (Lv et al., 2023), chilling in rice (Zhang et al., 2023) and onions (Hancı et al., 2019), and salinity in sorghum (Kiremit et al., 2024) and desert species (Zhang et al., 2024b). These outcomes suggest that MLT functions as a hub modulator in co‐priming strategies.

Co‐application of MLT's with metabolic signals γ‐aminobutyric acid in Cd‐stressed tomato seeds (Lv et al., 2023) or with L‐tryptophan in chilling‐exposed *Allium* species (Hancı et al., 2019) enhances seed vigor and radicle elongation more than individual treatments. These combinations suggest that metabolic priming with MLT boosts early energy metabolism and reserve mobilization, and coordinates detoxification responses by upregulating enzymatic antioxidants and limiting ion toxicity.

However, synergistic effects are dose‐dependent. For instance, the combined application of auxin with MLT results in a dual effect that depends on the concentration. Low doses (e.g., 100 μmol L^−1^) enhance growth and stress tolerance, but high doses (e.g., 200–300 μmol L^−1^) inhibit germination in desert species (Zhang et al., 2024b). This biphasic or “dual” effect reflects a concentration‐dependent shift from stimulation to inhibition. It indicates the importance of optimizing dosages and treatment durations to avoid antagonistic effects in hormonal crosstalk.

The mechanisms by which MSP enables stress tolerance (as discussed earlier and highlighted in Figure 4) are further strengthened by co‐priming examinations. For instance, MLT combined with SA or proline effectively limits Na^+^ uptake while boosting K^+^/Na^+^ and Ca^2+^/Na^+^ ratios, thus maintaining osmotic and nutrient homeostasis under salinity stress in sorghum (Kiremit et al., 2024). Likewise, when combined with GA_3_ or BR, MLT enhances root system architecture, metabolic activation, and the antioxidant defense network in cold‐stressed rice seedlings (Zhang et al., 2023). These findings improved our knowledge that MLT acts less as a solo protector and more as a synergistic mediator of stress‐responsive pathways.

### Nanoformulations

Integration of MLT into nanoformulations is considered a unique advance in seed priming strategies that offers extraordinary control over delivery, stability, and bioactivity (Mukherjee et al., 2024; Santos et al., 2025). Traditional MSP has proven effective in modulating stress responses; nano‐enabled MSP leverages the advantages of slow release, enhanced uptake, and targeted delivery, thereby uplifting MLT's potential to mitigate abiotic stresses.

An emerging finding across studies is that nano‐carriers not only preserve MLT's antioxidant integrity but also prolong its physiological effects, thus allowing sustained defense against stress‐induced oxidative bursts. For instance, chitosan‐ and silicon‐based MLT nanocarriers significantly enhanced ROS scavenging and redox balance under salinity in spearmint (Gohari et al., 2023) and under nickel stress in rice (Li et al., 2022), which clearly demonstrates an advantage over free MLT. This prolonged antioxidant activity is not merely passive buffering; it actively maintains plasma membrane integrity, modulates aquaporin expression (*PIP*), and sustains osmotic balance under salinity, as seen in tomato (Masoumi et al., 2024) and corn salad systems (Gohari et al., 2024b). Importantly, nanoparticle‐based MSP enables molecular‐level reprogramming, including the downregulation of stress‐induced metal transporter genes (e.g., *OsHMA2*, *OsHMA3*, *OsIRT1*, *OsIRT2*, *OsNramp1*, *OsNramp5*, and *OsLCT1*) in Cd‐stressed rice, thereby restricting toxic ion uptake and translocation (Jiang et al., 2021). This suggests that nano‐MLT does more than alleviate symptoms; it directly interferes with stress perception and signaling at the transcriptional level, a functional upgrade over classical priming approaches.

The superiority of nanoformulations also extends to enhanced physiological and metabolic tolerance. For example, nano‐MSP improved germination under nickel stress more effectively than free MLT by modulating reserve mobilization, phytohormone dynamics, and nutrient uptake (Li et al., 2022). Similarly, chitosan‐MLT nanoparticles enriched Chl content, phenolics, and essential secondary metabolites (catechins and o‐coumaric acid), as noticed against salinity stress in spearmint (Gohari et al., 2023) and corn salad (Gohari et al., 2024b), which linked stress tolerance with nutraceutical value.

The above argument supports the notion that nanoformulated MLT strengthens core MSP pathways, including ROS detoxification, proline accumulation, ion homeostasis (particularly K^+^/Na^+^ balance), and enhances antioxidant activities. However, nano‐delivery platforms intensify these responses by ensuring that MLT is delivered precisely when and where it is needed, whether during early imbibition, at root tips under salinity, or at foliar sites exposed to oxidative bursts.

From an application stance, seed coating with MLT‐loaded nanoparticles (e.g., chitosan, alginate, or silicon) provides a practical, scalable solution that combines storage stability with field viability. These coatings are important for synchronous germination, uniform seedling emergence, and consistent priming effects, traits that are critical for large‐scale arrangement under variable field conditions. In parallel, biopolymer‐based coatings such as HYDR‐MLT have also shown potential in delivering MLT in a slow‐release, eco‐friendly manner. An MLT‐embedded alginate hydrogel coating significantly improved germination parameters, root growth, and oxidative stress tolerance under salinity in tomato. The hydrogel system modulated key biochemical markers such as MDA and H_2_O_2_, enhanced seedling vigor, and demonstrated comparable functional benefits to nanoformulated MLT (Athanasiou et al., 2025). These findings suggest that natural polymer‐based formulations could be considered a green and scalable alternative to conventional nano‐carriers.

Some key challenges related to field efficacy, nanoparticle safety, and regulatory models should be addressed in future research. Long‐term effects on soil microbiota, plant–microbe interactions, and food safety need careful evaluation. In short, as agriculture moves toward climate‐smart solutions, nano/hydrogel‐enabled MLT (nanoMLT priming) delivery stands at the connection of nanotechnology, seed biology, and plant stress physiology (Mukherjee et al., 2024), which can redefine innovative priming strategies.

### Co‐application with beneficial microbes

MLT not only acts as a potent stress alleviator but also complements the plant growth‐promoting activities of beneficial microbes, particularly rhizobacteria and endophytes. When applied in combination, MLT and beneficial microbes co‐regulate redox balance, hormonal signaling, ion homeostasis, and nutrient uptake, thus improving plant growth, photosynthesis, and survival under abiotic stress. For instance, MSP, followed by *Rhizobium* inoculation, significantly improves salinity tolerance in common bean, by boosting antioxidant activity, maintaining K^+^/Na^+^ balance, and enhancing shoot biomass and seed yield (Alinia et al., 2022a; 2022b). The presence of ACC deaminase and IAA‐producing rhizobacteria further enables selective ion transport, nitrogen fixation, and mitigation of Na^+^ toxicity, mechanisms that are also potentiated by MLT through modulation of oxidative stress responses and protein synthesis (Alinia et al., 2022a; 2022b).

Field trials with faba bean demonstrated that combining MLT with exopolysaccharide‐producing *Azotobacter* and *Rhizobium* enhanced biomass, photosynthetic pigments, and yield, while reducing Na^+^ and Cl^−^ accumulation in salty soils (Abd El‐Ghany and Attia, 2020). These results highlight the potential of integrated MLT–microbe formulations for natural soil constraints where both osmotic and ionic stresses co‐exist.

Under drought, co‐application of MLT and *Lysinibacillus fusiformis* in soybean significantly increased endogenous MLT levels and enhanced antioxidant defense, JA/SA signaling, and nutrient (Ca, K, and Mg) uptake. This was accompanied by downregulation of ABA biosynthesis (*NCED3*) and upregulation of drought‐responsive TFs (DREB2, bZIP, and ERD1), which supported a multi‐hormonal and transcriptional regulatory model for stress tolerance (Imran et al., 2023). Similarly, under heat stress, MLT co‐applied with *Rhizobium* in *Medicago truncatula* enhanced nitric oxide production, nitrate reductase activity, photosynthesis, and antioxidant defenses, while reducing oxidative damage and lipid peroxidation (Irshad et al., 2022). These studies point to a multi‐tiered synergy, in which MLT augments microbial colonization and bioactivity, while microbes, in turn, enhance MLT uptake, signaling crosstalk, and systemic tolerance.

A recent study on tomato also confirmed that combining arbuscular mycorrhizal fungi with natural priming compounds (SA or chitosan) can modify the transcriptome and metabolome, which can enhance antioxidant and osmoprotective pathways under both drought and salinity stresses (Giovannini et al., 2024). This outcome further highlights that integrating biopriming and microbial symbiosis can harness synergistic physiological tolerance by coordinating molecular and metabolic adjustments in stressed plants.

Regardless of these advances, whether MLT selectively recruits beneficial microbial communities or alters root exudate profiles remains poorly understood. However, for symbiotic microbes such as mycorrhizae, MLT may exert an indirect influence by improving photosynthetic efficiency and photosynthate distribution, thus providing more carbon substrates to the fungal partner and promoting higher colonization and nutrient exchange efficiency (Xia et al., 2022; Ye et al., 2022). We argue that future work should focus on personalized consortia formulations, precision delivery systems (e.g., nanoMLT‐biopriming), and field‐scale validations. These outcomes can help fully harness the potential of MLT–microbe synergy for stress‐smart agriculture.

## MOLECULAR INTERACTION AND CROSSTALK UNDER ABIOTIC STRESS CONDITIONS: A FOCUS ON UNDEREXPLORED MELATONIN‐PRIMED SEEDS

It should be noted that foliar‐applied MLT crosstalk and interaction with other signaling molecules under abiotic stresses are well documented (Wang et al., 2018, 2024b; Raza et al., 2022; Zeng et al., 2022b; Ahmad et al., 2023; Colombage et al., 2023; Pan et al., 2023; Huang et al., 2024; Jindal et al., 2024; Rachappanavar, 2025; Sun et al., 2025). Nevertheless, the molecular interactions triggered by MSP remain underexplored. In contrast to foliar applications, MSP initiates early molecular reprogramming, which enables seeds to better sense and respond to stress. One essential feature of this priming effect is hormonal crosstalk between MLT and ABA. In multiple crops, including cotton (Xiao et al., 2019), cucumber (Zhang et al., 2014), soybean, wheat, maize, and alfalfa (Luo et al., 2024), MSP decreased ABA content by downregulating biosynthesis genes (e.g., *NCED3*, and *NECD2*) and enhancing catabolism genes (e.g., *CYP707A1*/*2*), while simultaneously increasing the biosynthesis of gibberellins (GA_3_ and GA_4_). Notably, low doses of MLT (5, 50, and 100 μM) were optimal for enhanced seed germination, antioxidant enzyme activity, and reduced MDA accumulation under cold stress in maize, onion, and leek (Cao et al., 2019; Hancı et al., 2019), whereas higher MLT levels (200 μM) led to loss of these effects or became inhibitory in cotton, which highlights a concentration‐dependent hormonal balance (Xiao et al., 2019). This hormonal rebalancing was closely linked with MLT‐induced H_2_O_2_ accumulation, which acted as a second messenger to promote ABA catabolism and GA synthesis, as noticed in Arabidopsis (Li et al., 2021a). These GA–ABA shifts were also observed when MSP was combined with GA_3_ priming in maize under drought stress (Kumari et al., 2026) and with SA in sunflower under salinity (Zia et al., 2026), suggesting MLT's role as a hub in multi‐hormonal crosstalk. Notably, calcium signaling via *CAX3*‐mediated Ca^2+^ efflux was also identified as a key player in this redox–hormone interplay (Li et al., 2021a). Together, these processes explain how MSP enhances seed germination and early vigor under multiple stresses.

Moreover, MSP also activates broader stress signaling networks. For example, in cotton, Supriya et al., (2024) demonstrated that MSP enhanced autophagy under drought by activating ABA‐independent MAPK–SnRK2–SnRK1 signaling, and modulated key regulators like *ATG8*, *RAPTOR1*, *TPS63*, and *TPP22*. In rapeseed, MSP with GA_3_ improved drought tolerance by promoting antioxidant enzymes and proline accumulation, and linked hormonal synergy with redox buffering and osmoprotection under both controlled and field conditions (Khan et al., 2020). Likewise, in common bean, MSP improved the antioxidant system, Na^+^/K^+^ balance, and photosynthetic activity, and interacted synergistically with *Rhizobium* to boost salinity tolerance (Alinia et al., 2022b). These effects were also evident in sorghum, where MSP enhanced ion homeostasis and macro/micronutrient uptake under salinity (Kiremit et al., 2024). Meanwhile, in rice (Ma et al., 2025), desert plants (Zhang et al., 2024b), and sunflower (Zia et al., 2026), co‐priming with ABA, IAA, SA, or proline showed preservative benefits by converging on antioxidant activity, osmotic balance, and germination efficiency.

Moreover, MSP also fine‐tunes redox regulation and osmotic adjustment via synergistic interaction with other molecules. For instance, in onion and leek under temperature stress (Hancı et al., 2019) and desert species under salinity (Zhang et al., 2024b), MSP modulated antioxidant enzyme activities, MDA levels, and proline accumulation in a dose‐sensitive manner. In maize, MSP improved chilling stress tolerance by enhancing germination indices and antioxidant enzyme activity, while also boosting starch metabolism, a vital energy source during early growth under cold (Cao et al., 2019).

The molecular crosstalk hubs and downstream targets remain underexplored, especially at the proteomic and ionomic levels. Harnessing these pathways can open new frontiers in seed priming biology and help design targeted strategies for climate‐resilient agriculture.

## HARNESSING MODERN MOLECULAR TOOLS WITH MELATONIN‐PRIMED SEEDS: A PERSPECTIVE FOR NEXT‐GENERATION, STRESS‐SMART SEEDS/PLANTS

**Figure 7:**
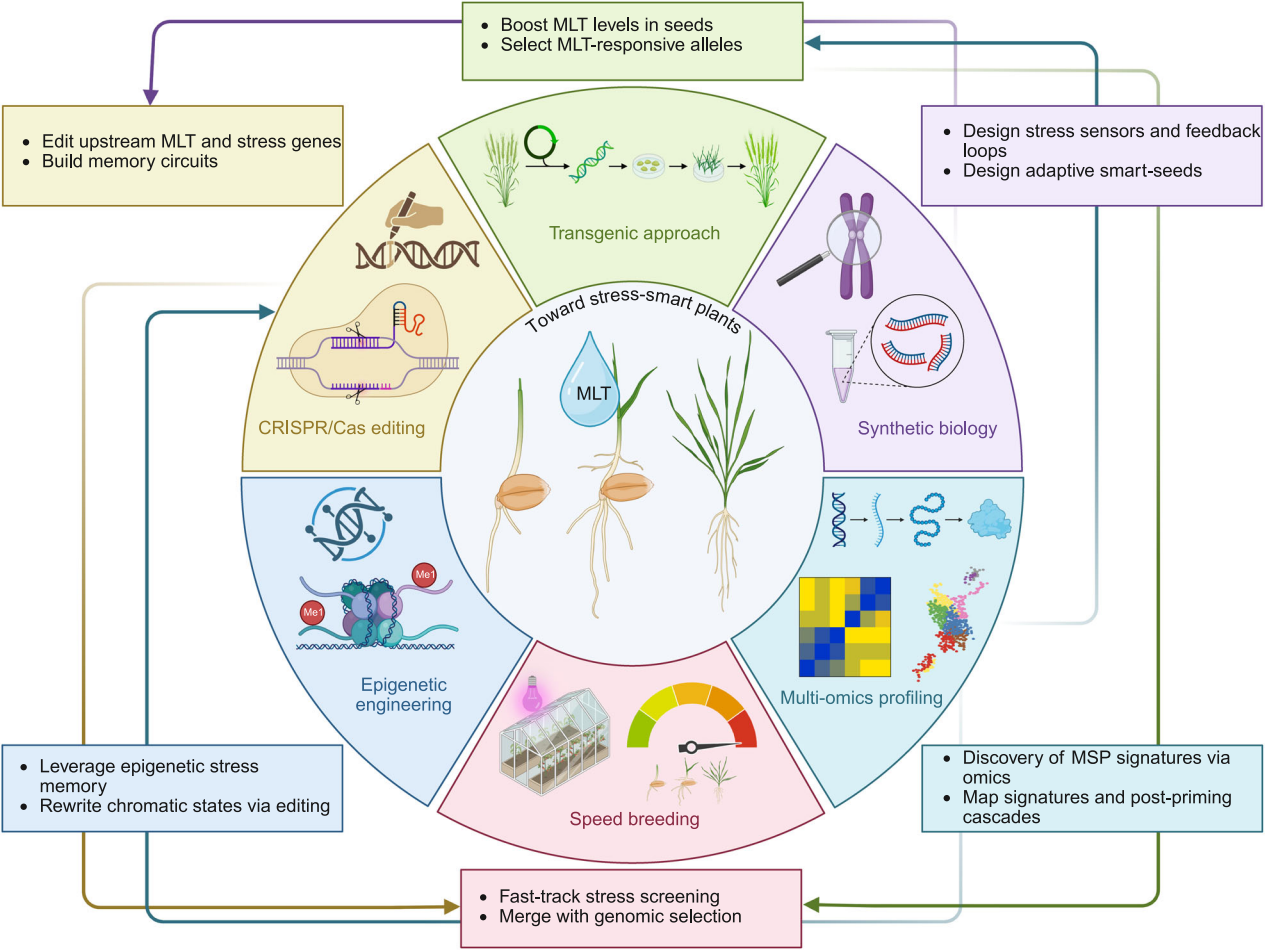
Integrative roadmap of advanced molecular and breeding tools with melatonin‐primed seeds for designing next‐generation, stress‐smart crop plants Arrows indicate the integration of one tool into another. Created in https://BioRender.com. CRISPR/Cas, clustered regularly interspaced short palindromic repeats/CRISPR‐associated proteins; MLT, melatonin; and MSP, melatonin seed priming.

Integrating MSP with modern molecular breeding and omics‐driven platforms is an innovative and forward‐looking step toward developing stress‐smart plants (Figure 7). The transient nature of the MSP‐induced primed state raises a fundamental question: Can this biochemical advantage be stabilized, genetically encoded, or even mimicked through targeted breeding or engineering? The answers remain unclear, but emerging genomic tools offer powerful avenues to explore this question and, in doing so, dissect the still‐undefined molecular basis of MSP. Here, we propose a perspective on how such tools could be explored to transform MSP from a descriptive phenomenon into a mechanistically understood and genetically tractable trait.

A logical starting point is to ask whether the benefits of MSP can be recapitulated by enhancing endogenous MLT production. Transgenic studies provide strong proof of concept, demonstrating that overexpression of key MLT biosynthetic genes, such as *COMT* in Arabidopsis, orange, tomato, rose, watermelon, and rice (Liu et al., 2019; Li et al., 2024, 2025; Xu et al., 2024; Guo et al., 2025; Zhu et al., 2025a); *SNAT* in Arabidopsis and cotton (Wu et al., 2021; Zhang et al., 2022); and *ASMT* in tobacco, apple, and poplar (Yu et al., 2022, 2025; Gao et al., 2024), confers enhanced tolerance against multiple stresses (including drought, salinity, heavy metals, nitrogen deficiency, and temperature). The resulting phenotypes are mainly facilitated by hormonal modulation, potent ROS scavenging, and the accumulation of protective osmolytes. Similar findings are observed in MSP‐treated seeds (as detailed in earlier sections on stress types). This raises a key, testable hypothesis that genetic variants (alleles) with elevated basal or inducible MLT levels in seeds could be identified and selected for, potentially mimicking the priming effect without exogenous application. Exploring natural variation in MLT biosynthesis and signaling pathways is a critical first step toward integrating MSP responsiveness into breeding programs.

Another major bottleneck is our profound ignorance of the molecular signature specific to MSP. Over the past decade, different omics approaches have advanced plant stress biology (Derbyshire et al., 2022; Joshi et al., 2024; Dobránszki et al., 2025; Raza et al., 2025b, 2026a; Ateeq et al., 2026; Kundu and Tanti, 2026); they have been narrowly applied within the MSP context. Furthermore, unanswered questions include the following: What are the temporal transcriptomic and metabolomic changes that uniquely define the primed seed during the post‐germination phase (Balmer et al., 2015; Srivastava et al., 2021; Macovei et al., 2025)? Do post‐translational modifications, identifiable by proteomics, sustain the primed state? Can ionomics guide how MSP fine‐tunes nutrient homeostasis during early development? Addressing these questions through targeted, hypothesis‐driven omics experiments is not an aspirational goal. Instead, it is a prerequisite for moving forward toward a mechanistic understanding (Dobránszki et al., 2025).

The hypothetical role of epigenetic regulation in long‐term MSP effects is a particularly interesting, yet entirely speculative, area. Studies in related fields suggest that priming effects may involve heritable epigenetic marks, such as DNA methylation, histone modifications, or small RNAs (Cañizares et al., 2025; Dobránszki et al., 2025; Macovei et al., 2025). Nevertheless, direct findings for such mechanisms following MSP are completely absent. Therefore, we suggest that future work must ask the following: Does MSP induce persistent epigenetic reprogramming, or are its effects purely transient and biochemical? Techniques such as small RNA profiling and bisulfite sequencing are now available to test this hypothesis, which will help discover whether MSP leads to persistent reprogramming or reversible changes. If a stable epigenetic signature of MSP‐induced stress memory is confirmed, these marks could be harnessed as predictive biomarkers or targeted via epigenome editing (Lloyd and Lister, 2022; Cheng et al., 2024; Dobránszki et al., 2025).

Likewise, genome editing tools such as CRISPR/Cas are not intended for immediate application, but for hypothesis testing (Kumlehn et al., 2018; Li et al., 2023; Zaman et al., 2024; Chen et al., 2026). Rather than claiming that they will create MSP‐mimicking crops, we propose that these tools can frame the following question: Do specific upstream regulators of MLT biosynthesis or ROS signaling control the priming response? (Zhu et al., 2025a). Can transient activation of defense genes via CRISPRa during germination simulate the MSP effect (Ding et al., 2022)? Answering these causal questions in the laboratory is a necessary precursor to any translational application.

Synthetic biology tools can enable the design of stress‐inducible or synthetic memory circuits that respond to seed‐stage cues and enable the engineering of “smart” seeds with programmable priming responses (Kumlehn et al., 2018; Roell and Zurbriggen, 2020; Lloyd et al., 2022; Yang and Reyna‐Llorens, 2023).

High‐throughput platforms such as speed breeding and genomic selection are not presented here as ready‐to‐use pipelines but rather as envisioned methods for future integration (Gao et al., 2025; Wang et al., 2026b). The challenge will first be to identify physiological or transcriptional markers that reliably predict a genotype's responsiveness to MSP. Once such markers are validated, they could, in principle, be used to accelerate the selection of priming‐compatible cultivars within a speed‐breeding context (Watson et al., 2018; Fu et al., 2025; Wang et al., 2026b).

Our above discussion does not claim that MPS is ready for integration with advanced molecular tools. Rather, we argue that the most emerging questions in the MSP field (e.g., its molecular, stability, and genetic basis) cannot be answered without them. As illustrated in Figure 7, integrating MSP with high‐throughput tools could redefine the field from documenting the effects of MSP to understanding its mechanisms, and ultimately, to evaluating its true potential for crop improvement.

## CHALLENGES AND BOTTLENECKS ASSOCIATED WITH MELATONIN SEED PRIMING: FINDING NEW RESEARCH TARGETS

**Figure 8:**
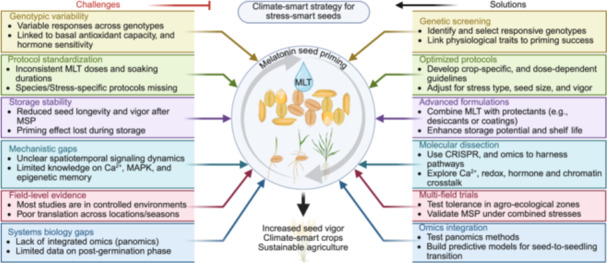
Overview of the major challenges and future directions associated with melatonin seed priming The left half of the diagram summarizes key bottlenecks, while the right half describes potential solutions. This infographic highlights the translational challenges that must be addressed to harness the full potential of melatonin seed priming for climate‐smart seed technologies. Created in https://BioRender.com. Ca^2+^, calcium ion; MAPK, mitogen‐activated protein kinase; MLT, melatonin; and MSP, melatonin seed priming.

Regardless of its protective role in stress management, MSP faces several critical challenges that hinder its wide‐scale implementation and mechanistic understanding (Figure 8). Building on the limitations and patterns identified in section “A CRITICAL ASSESSMENT OF MSP: LIMITATIONS, INCONSISTENCIES, AND KNOWLEDGE GAPS”, these challenges define key priorities for underexplored research and translational development.

One major limitation is the high specificity of priming responses to genotype. Responses vary noticeably even within species, largely reflecting differences in basal ROS levels, antioxidant potential, and hormonal sensitivity. This variability necessitates genotype‐aware optimization strategies rather than universal priming protocols, particularly for crops with high genetic diversity.

Protocol optimization, particularly dose and treatment duration, is another unresolved issue. As systematically analyzed in Figure 6, MSP shows clear hormetic behavior, with benefits concentrated at low to moderate doses and inhibitory effects at higher doses. Soaking time and seed characteristics further influence outcomes; yet, standardized, stress‐ and species‐specific protocols are still lacking. Importantly, the near absence of multi‐factorial dose–response studies under combined stresses represents a major gap, limiting the development of strong and field‐relevant MSP strategies.

Post‐priming seed storage stability poses a practical barrier for commercial adoption. Primed seeds frequently lose vigor during prolonged drying and storage due to reduced desiccation tolerance, narrowing the safe sowing window. Addressing this limitation will require the development of stabilized delivery systems (e.g., protective coatings and nano‐ or hydrogel‐based formulations) that preserve the primed state without compromising seed longevity.

Most MSP studies rely on controlled lab or greenhouse conditions, with limited multi‐location field validation and scarce data on yield stability under natural multifactorial stresses. Interactions with standard seed‐industry practices and economic/regulatory aspects remain largely unaddressed. Bridging this gap will require coordinated multi‐year, multi‐location field trials that integrate MSP within existing agronomic and industrial models, rather than evaluating it in isolation.

At the mechanistic level, while core pathways such as ABA–GA–ROS crosstalk are increasingly being documented, the precise spatiotemporal dynamics of downstream signaling modules, autophagy regulation, and potential epigenetic contributions during the early seedling developmental transition remain to be elucidated. In particular, distinguishing MSP‐specific regulatory networks from those inferred from foliar MLT applications remains a critical unresolved issue. Multi‐omics integration focused specifically on early post‐germination phases remains scarce, which largely hinders the construction of predictive regulatory networks.

At the experimental and comparative levels, future investigations must also prioritize in‐depth study design, transparent reporting of seed‐lot characteristics, and direct comparisons with established priming approaches to determine the true benefit of MSP.

## CONCLUSION AND FUTURE RECOMMENDATIONS

MSP is still emerging as a powerful green priming strategy that enhances plant stress tolerance by activating key physio‐biochemical and molecular pathways from the seed stage onward. It has demonstrated substantial but context‐dependent benefits across various abiotic stresses through integrated physio‐biochemical and molecular reprogramming (see Table 1 for detailed insights). Given that seeds are the foundation of agriculture (both the primary agricultural input and a critical nutritional output), the integration of MSP into the seed sector suggests a direct pathway to produce stress‐smart seeds that retain high vigor, germination potential, and nutritional quality even under challenging climatic conditions. The global seed market, projected to exceed $100 billion by 2030 (https://www.mordorintelligence.com/industry-reports/seeds-industry), is increasingly driven by demand for climate‐smart varieties; MSP can directly support this need by delivering potentially commercially viable, climate‐smart seed‐lots to farmers at large scales. Overall, we conclude that MLT is a low‐cost natural compound that is particularly attractive for sustainable and stress‐smart agriculture. However, its large‐scale positioning will depend on resolving key biological and translational constraints.

Though specific MLT concentrations and stress levels are summarized in Table 1, we discussed mechanistic insights rather than individual numeric values throughout the review, as these factors differ greatly across species, stress types, developmental stages, and experimental systems. Thus, future work should prioritize standardized dose–response models across species and stress conditions. We anticipate that with the help of universal dose–response systems, we will be able to compare results across studies, precisely model hormetic thresholds, and optimize MLT‐based priming protocols for practical use. Particularly, future efforts should prioritize examining genotype × environment × dose interactions and expand these assessments to combined stress conditions, which remain largely underexplored. Therefore, we suggest that future meta‐ and systems‐level investigations should clearly address these interdependencies to define dose‐specific guidelines for both MSP and exogenous applications under both single and combined stress conditions.

**Figure 9:**
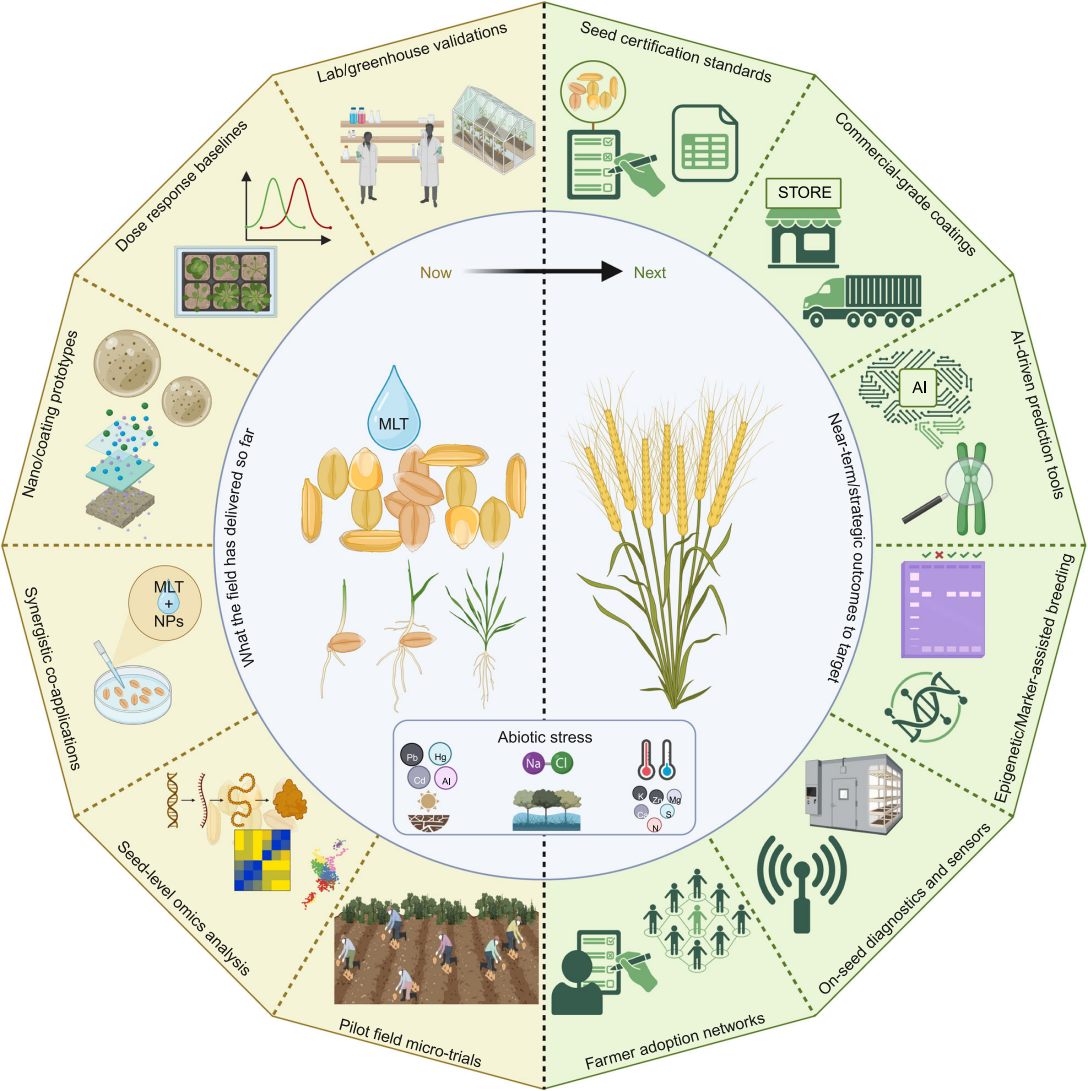
Systematic ways for moving melatonin seed priming from laboratory proofs to scalable climate‐smart agriculture The circular panel demonstrates the transition from current experimental achievements (left‐yellow) to targeted near‐term outcomes (right‐green). Created in https://BioRender.com. AI, artificial intelligence; MLT, melatonin; and NPs, nanoparticles.

Despite substantial progress, key challenges hinder the large‐scale application of MSP (Figure 8). Moreover, critical stress domains such as light stress, nutrient deficiency, and elevated CO_2_ or ozone exposure remain largely untested under MSP, which signifies major research gaps that need urgent attention. We also suggest that upcoming work address seed storage limitations post‐priming, protocol standardization across crops, and the logistics of integrating MSP into seed supply chains. Importantly, bridging the gap between controlled‐environment findings and field‐scale performance remains a key priority for validating MSP as a truly climate‐smart, sustainable solution. These challenges and actionable solutions are synthesized in the translational model; Figure 9 provides a detailed breakdown of current achievements, technological gaps, and targeted future outcomes.

Modern technologies must be exploited to harness the full potential of MSP (see Figure 7 and related text for detailed arguments). In parallel, innovation in formulation and delivery systems, such as encapsulation, seed coatings, or nanocarriers, can extend shelf‐life, improve uptake, and ensure farmer‐ready deployment of MSP technologies. Advances in materials science and bioengineered seed‐coating technologies (e.g., biodegradable hydrogel matrices and nanoclay composites) could enable controlled MLT release, protect it from environmental degradation, and extend the shelf‐life of primed seeds. We also suggest that the integration of these innovations with AI‐guided optimization and synthetic biology tools may further pave the way for next‐generation, precision seed treatment systems (Fu et al., 2025; Sanchez‐Munoz and Roig‐Villanova, 2025; Zhu et al., 2025c; Wang et al., 2026b). Moreover, field‐scale trials are urgently needed to evaluate performance under natural constraints, and cost–benefit analyses must be conducted to demonstrate economic viability. Equally importantly, compatibility with existing seed‐industry practices and regulatory considerations must be systematically evaluated. As depicted in Figure 9, the strategic vision for MSP extends from reproducible laboratory proofs to scalable, certified, farmer‐led adoption networks that provide a clear scheme for transitioning this technology into the global seed industry. Ultimately, the way forward lies in integrating MSP with precision agriculture, molecular breeding, and agronomic best practices, supported by clear regulatory structures and extension services. When effectively validated and integrated into commercial seed production pipelines, MSP has the potential to become a major enabler of the next generation of stress‐smart, high‐vigor seeds, readily available to farmers worldwide. With coordinated global research efforts, MSP can evolve from a beneficial laboratory tool into a cornerstone of future‐ready, climate‐resilient crop production systems. Moving forward, addressing these gaps requires a set of focused, high‐impact research directions. The questions that remain are as follows:

1. How can crop‐ and genotype‐specific dose–response baselines for MSP be established across diverse cultivars and stress conditions?
2. What molecular and epigenetic signatures define long‐term MSP effects from seed maturity?
3. Can MSP‐driven multi‐omics integration (panomics) deliver universal and species‐specific MSP mechanisms?
4. How can genotype × environment × dose interactions be leveraged and predicted for scalable MSP exploitation?
5. Can AI‐driven predictive models fast‐track the optimization of MSP for field conditions and stress combinations?
6. How can nanotechnology and biodegradable coatings be harnessed for precise MLT delivery in commercial seed systems?
7. Can marker‐assisted or CRISPR‐based breeding target alleles that boost crop responsiveness to MSP?
8. What farmer‐centered networks and seed certification structures are required to incorporate MSP into climate‐smart agriculture?

## CONFLICTS OF INTEREST

The authors declare no conflicts of interest.

## AUTHOR CONTRIBUTIONS

Z.H., V.F., and A.R., conceived the idea. A.R. prepared the original draft and designed the figures and tables with inputs from L.Y. C.G. E.K., E.A., M.J., J.Z., V.F., and Z.H. critically reviewed, provided insightful suggestions, and edited the manuscript. All authors have read and approved the final version of the manuscript.
